# The Relationship among Tyrosine Decarboxylase and Agmatine Deiminase Pathways in *Enterococcus faecalis*

**DOI:** 10.3389/fmicb.2017.02107

**Published:** 2017-11-01

**Authors:** Marta Perez, Victor Ladero, Beatriz del Rio, Begoña Redruello, Anne de Jong, Oscar Kuipers, Jan Kok, M. Cruz Martin, Maria Fernandez, Miguel A. Alvarez

**Affiliations:** ^1^Dairy Research Institute, Consejo Superior de Investigaciones Científicas (IPLA-CSIC), Asturias, Spain; ^2^Department of Molecular Genetics, Groningen Biomolecular Sciences and Biotechnology Institute, University of Groningen, Groningen, Netherlands

**Keywords:** *Enterococcus faecalis*, putrescine, tyramine, biogenic amines, regulation, biosynthesis

## Abstract

Enterococci are considered mainly responsible for the undesirable accumulation of the biogenic amines tyramine and putrescine in cheeses. The biosynthesis of tyramine and putrescine has been described as a species trait in *Enterococcus faecalis.* Tyramine is formed by the decarboxylation of the amino acid tyrosine, by the tyrosine decarboxylase (TDC) route encoded in the *tdc* cluster. Putrescine is formed from agmatine by the agmatine deiminase (AGDI) pathway encoded in the *agdi* cluster. These biosynthesis routes have been independently studied, tyrosine and agmatine transcriptionally regulate the *tdc* and *agdi* clusters. The objective of the present work is to study the possible co-regulation among TDC and AGDI pathways in *E. faecalis*. In the presence of agmatine, a *positive* correlation between putrescine biosynthesis and the tyrosine concentration was found. Transcriptome studies showed that tyrosine induces the transcription of putrescine biosynthesis genes and up-regulates pathways involved in cell growth. The tyrosine modulation over AGDI route was not observed in the mutant Δ*tdc* strain. Fluorescence analyses using *gfp* as reporter protein revealed P*aguB* (the promoter of *agdi* catabolic genes) was induced by tyrosine in the wild-type but not in the mutant strain, confirming that *tdc* cluster was involved in the tyrosine induction of putrescine biosynthesis. This study also suggests that AguR (the transcriptional regulator of *agdi*) was implicated in interaction among the two clusters.

## Introduction

Species belonging to *Enterococcus* genus are part of the cheeses microbiota, where they can reach 10^5^ to 10^7^ colony forming units (cfu) g^-1^ in the final product. These bacteria are present in the milk and are mainly found in traditional cheeses produced with raw milk. However, enterococci can also accumulate in cheeses elaborated with pasteurized milk as a consequence of contaminations during the fabrication course (Giraffa, 2003). Moreover, it has been observed that pasteurization does not completely eliminate them (Ladero et al., 2011).

Enterococci contribute to the cheese maturation, participating in the organoleptic properties development. Furthermore, they comprise interesting biotechnology features such as lipolytic activities, citrate utilization, volatile compounds biosynthesis, and bacteriocin production. Indeed, some *Enterococcus faecalis* strains with ability to produce bacteriocins have been proposed as adjunct cultures for food preserving (Giraffa, 2003).

Nevertheless, several authors have found a relation between the enterococci amounts in cheeses and the concentrations of tyramine (Burdychova and Komprda, 2007; Fernandez et al., 2007; Bonetta et al., 2008; Ladero et al., 2010b) and putrescine (Ladero et al., 2012a). Therefore, enterococci are considered mainly responsible for the undesirable accumulation of the biogenic amines (BAs) tyramine and putrescine in cheeses (Linares et al., 2012).

The consumption of foods with high concentrations of tyramine can cause intoxications. In fact, tyramine is responsible of the “cheese effect” (ten Brink et al., 1990) which involves symptoms as migraines and hypertension and can even cause cerebral hemorrhages (Ladero et al., 2010a; EFSA, 2011; Pessione, 2012). Tyramine citotoxicity has been recently demonstrated *in vitro*, indicating that at concentrations frequently encountered in BA-rich food, this BA produces necrosis in intestinal cells (Linares et al., 2016). In the same way, tyramine shows a cytotoxic synergistic effect with histamine – a common BA also found in cheeses (Del Rio et al., 2017). Although non-toxic effects have been directly related with diet putrescine, this BA could have a role in promoting some types of cancer (Alvarez and Moreno-Arribas, 2014) and is involved in the nitrosamines formation in foods, which have a well-known carcinogenic effect (Drabik-Markiewicz et al., 2011).

The production of unwanted BA has been studied in *Enterococcus* from food, human and clinical origin, identifying tyramine or putrescine producing strains among different species (Ladero et al., 2009, 2012b; Jimenez et al., 2013). Furthermore, the biosynthesis of tyramine and putrescine has been described as a species level trait in *E. faecalis* (Ladero et al., 2012b).

**FIGURE 1:**
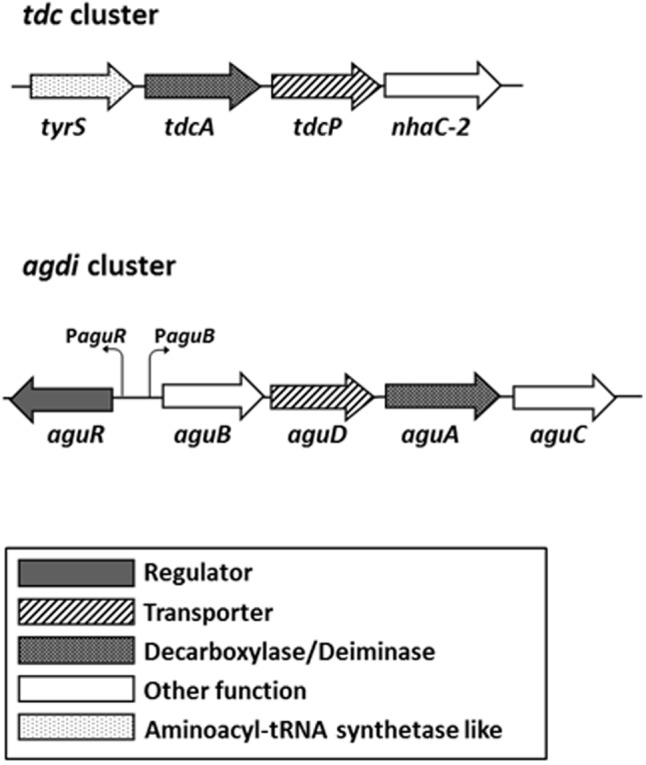
Genetic organization of the tyrosine decarboxylase (*tdc*) and agmatine deiminase (*agdi*) clusters in *Enterococcus faecalis*. The promoters of the *aguR* gene and *aguBDAC* operon are indicated. Adapted from Linares et al. (2014) and Ladero et al. (2017).

Tyramine is formed by the decarboxylation of the amino acid tyrosine, which exerts a role in the maintaining of the pH homeostasis in *E. faecalis* (Perez et al., 2016). The tyrosine decarboxylase (TDC) route is encoded in the *tdc* cluster, which comprises four genes (**Figure 1**). The catabolic genes *tdcA, tyrP*, and *nhaC-2* are co-transcribed as a polycistronic mRNA which expression is induced by tyrosine concentrations and acidic pH (Perez et al., 2015).

In *E. faecalis*, putrescine is formed from agmatine by the agmatine deiminase (AGDI) pathway, which is repressed by carbon source, suggesting a role in the energy production (Suarez et al., 2013). Five genes grouped in the *agdi* cluster are responsible of its biosynthesis: the regulator gene *aguR* and the metabolic genes *aguB, aguD, aguA*, and *aguC* (**Figure 1**). *aguR* is constitutively transcribed as a monoscistronic mRNA from its promoter P*aguR* in a very low expression level, while the catabolic genes are co-transcribed in a single mRNA from the *aguB* promoter (P*aguB*) in a divergent orientation (Linares et al., 2014). Furthermore, increasing concentrations of the substrate, agmatine, increase the transcription of the catabolic genes through the transmembrane regulator AguR (Suarez et al., 2013; Linares et al., 2014). Linares et al. (2015) suggest that AguR is a one component transcriptional activator that senses agmatine concentrations in the extracellular environment activating the transcription of the *aguBDAC* operon through its interaction with P*aguB*.

Although both *E. faecalis* BA biosynthesis routes have been extensively studied independently, it is unknown whether there is a relationship between them. Therefore, the objective of this work was to examine the potential co-regulation among TDC and AGDI catabolic pathways in *E. faecalis.* First, we considered whether the amino acid substrate of one route had any effect on the other. Once it was verified that in the presence of tyrosine, the biosynthesis of putrescine increased, we studied the responsible mechanisms of this putative modulation through a global analysis of the gene expression in the presence and absence of tyrosine, and the putative role of the genes involved in the biosynthesis of these BA.

## Materials and Methods

### Bacterial Strains and Growth Conditions

Two strains were used in this study: the wild-type *E. faecalis* V583 (Sahm et al., 1989) (hereafter referred to as wt) which biosynthesizes tyramine and putrescine (Ladero et al., 2012b) and the derived non-tyramine-producing mutant *E. faecalis* V583 Δ*tdc* (hereafter referred to as Δ*tdc*) that lacks tyrosine decarboxylase genes cluster (Perez et al., 2015). Bacteria were grown in M17 medium (Oxoid, Hampshire, United Kingdom) supplemented with 5 g L^-1^ glucose (Merck, Darmstadt, Germany) (GM17) at 37°C under aerobic conditions. Tyrosine, agmatine, or tyramine (Sigma–Aldrich, St. Louis, MO, United States) were added at indicated concentrations. Chloramphenicol (5 μg ml^-1^) (Sigma–Aldrich) was added when required. All cultures were inoculated at 0.1% (v/v).

### Plasmid and Bacteria Transformation

**FIGURE 2:**
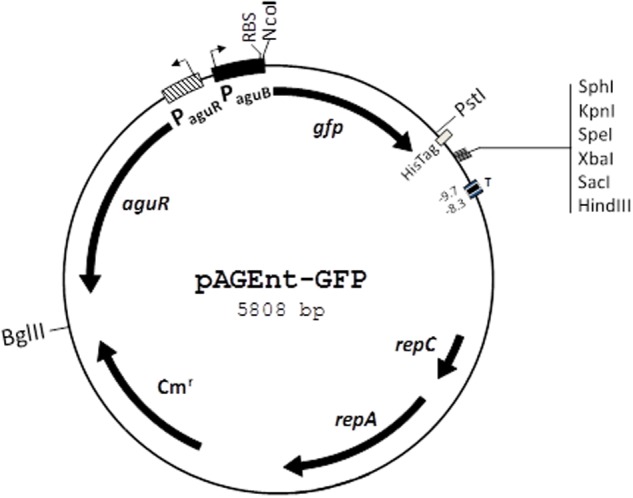
Genetic map of the pAGEnt-GFP vector. *repC* and *repA*, replication genes; Cm^r^, chloramphenicol resistance gene; *aguR*, gene encoding the regulatory protein AguR; P*aguR, aguR* promoter; P*aguB, aguBDAC* promoter; RBS, ribosome binding site; *gfp*, gene encoding green fluorescent protein; His-Tag, C-terminal histidine tag; T, transcription terminator. Restriction sites are indicated. Adapted from Linares et al. (2014).

The agmatine-inducible plasmid pAGEnt-GFP (**Figure 2**) carries P*aguR*, the *aguR* gene, and P*agu*B of *E. faecalis* V583 cloned to the promoterless gene *gfp* encoding green fluorescent protein (GFP) (Linares et al., 2014). pAGEnt-GFP was transformed into electrocompetent *E. faecalis* cells obtained as described previously (Perez et al., 2015).

### Quantification of Biogenic Amines

Supernatants obtained from centrifuged cultures were filtered through 0.2 μm polytetrafluoroethylene (PTFE) filters (VWR, Barcelona, Spain). BA were derivatized with diethyl ethoxymethylenemalonate (DEEMM) (Sigma–Aldrich). 100 μl of sample were mixed with 175 μl of 1 M borate buffer (1 M boric acid neutralized with NaOH until pH 9.0), 75 μl of methanol (Merck), 2 μl of L-2-aminoadipic acid as internal standard (2 g/L) (Sigma–Aldrich) and 3 μl of DEEMM. The mixture was incubated at 30°C in an ultrasound bath (Selecta, Barcelona, Spain) for 45 min. Samples were then heated at 70°C for 2 h to allow the complete degradation of excess DEEMM and reagent by-products. Samples were filtered through 0.2 μm PTFE membranes (VWR) before injection into the chromatograph system. Samples were diluted, when necessary, with 0.1 N HCl (Merck). BA were separated and quantified by ultra high performance liquid chromatography (UHPLC) system (Waters, Milford, MA, United States) with an UPLC^®^BEH C18 1.7 μm column (Waters), following the method previously described (Redruello et al., 2013). Empower 2 software (Waters) was used to control the system and to analyze the data. Standards were prepared with agmatine, putrescine dihydrochloride (Acros Organics, Geel, Belgium), tyrosine and tyramine in Milli-Q water. The BA concentrations provided are the average of three independent cultures.

### DNA Microarrays and Data Analysis

DNA microarrays of *E. faecalis* V583 (Agilent Technologies, Santa Clara, CA, United States) were designed using the Agilent eArray (v5.0) program according to the manufacturer’s instructions. Each microarray (8 K × 15 K) was designed to contain spots of two different 60-mer oligonucleotide probes (in duplicate) specific for each of the 3182 coding DNA sequences (CDSs) representing the chromosomal genes of the *E. faecalis* V583 genome (GenBank accession no. AE016830) (Paulsen et al., 2003). The design and disposition of the probes in the array were deposited in Gene Expression Omnibus (GEO) database (Platform GPL21449).

Total RNA was isolated from 30 ml of *E. faecalis* wt strain cultures grown in GM17 supplemented with 5 mM agmatine and in the presence or absence of 15 mM tyrosine until the end of the exponential phase of growth (7 h), as described by Perez et al. (2016). cDNA was synthesized from 20 μg of RNA using the SuperScript III reverse transcriptase kit (Life Technologies, Bleiswijk, The Netherlands). Then, 20 μg of cDNA were labeled with DyLight 550 or DyLight 650 dyes using the DyLight amine-reactive dyes kit (Thermo Scientific, Amsterdam, Netherlands). 300 ng of DyLight 550- and 300 ng of DyLight 650-labeled cDNA were mixed and hybridized for 17 h at 60°C in the *E. faecalis* V583 DNA microarray using the *In situ* Hybridization Kit Plus, the Hybridization Gasket Slide and the Agilent G2534 A Microarray Hybridization Chamber (Agilent Technologies) following the manufacturer’s indications, as previously described (Perez et al., 2016). Slides were scanned using a GenePix 4200 A Microarray Scanner (Molecular Devices, Sunnyvale, CA, United States) and images were analyzed using GenePix Pro v.6.0 software. Background subtraction and locally weighted scatterplot smoothing normalization were done using the standard routines provided by GENOME2D software available at http://genome2d.molgenrug.nl/index.php/analysis-pipeline. DNA microarray data were obtained from two independent biological replicates and one technical replicate (including a dye swap). Expression ratios were calculated from the comparison of four spots per gene per microarray (total of 20 measurements per gene). A gene was considered differentially expressed when a *p-*value of at least 0.001 was obtained and the expression fold-change was at least |2|. The microarray data were deposited in GEO database under the Accession no. GSE97219. Functional analysis of the genes differentially expressed was performed using Gene Set Enrichment Analysis (GSEA) using routines provided by GENOME2D software available at http://server.molgenrug.nl/index.php/gsea-pro.

### Quantification of Gene Expression by Reverse Transcription Quantitative PCR (RT-qPCR)

Gene expression of *aguA* gene was measured by RT-qPCR in cultures of the strains wt and Δ*tdc*. First, bacteria from cultures adjusted to a cell density of approximately 2 (Abs_600_) were harvested by centrifugation at 4°C and total RNA was extracted using TRI reagent (Sigma–Aldrich) as previously described (Linares et al., 2009). RNA samples (2 μg) were treated with 2 U of DNase I (Fermentas, Madrid, Spain) for 2 h, in order to eliminate any genomic contamination. Total cDNA was synthesized from 0.5 μg of RNA using the reverse transcription iScript^TM^ cDNA Synthesis kit (Bio-Rad Madrid, Spain). Gene expression was performed in a 7500 Fast real-time PCR System (Applied Biosystems, Carlsbad, CA, United States). After fourfold dilution of cDNA sample, 5 μl were mixed with 12.5 μl of SYBR Green PCR Master Mix (Applied Biosystems), 1 μl of each primer at 700 nM and 5.5 μl of RNase-free water. The primers aguAqF and aguAqR (Linares et al., 2014), which are specific for *aguA* gene of *E. faecalis*, were used. The expression of *recA* (the recombinase A encoding gene) and *tufA* (the elongation factor thermo-unstable encoding gene) were used as internal controls to normalize the RNA concentration, using the specific primers recAF and recAR, and EFV583-tufF and EFV583-tufR, respectively (Linares et al., 2014). Amplifications were performed using the default cycling settings established by Applied Biosystems. Gene expression was calculated following the 2^-ΔΔC_T_^ method (Livak and Schmittgen, 2001). RT-qPCR analysis was performed on RNA purified from three biological replicates for each condition.

### Green Fluorescence Measurements

Total fluorescence of wt and Δ*tdc* cells harboring pAGEnt-GFP vector was measured following previous indications (Linares et al., 2014). Briefly, cells from cultures adjusted to a cell density 2 (Abs_600_) were harvested, washed in 400 μl of 50 mM potassium phosphate buffer pH 7.2 and resuspended in 200 μl of the same buffer. GFP emission was determined using a Cary Eclipse fluorescence spectrophotometer (Varian, Inc., Palo Alto, CA, United States), at 485 and 530 nm of excitation and emission wavelengths, respectively. Background fluorescence levels were assessed by measuring non-fluorescent control cells and subtracting the obtained values.

### Statistical Analysis

Results were presented as means and standard deviations. Statistical analyses were performed with the software SPSS version 15.0 (SPSS, Inc., Chicago, IL, United States) using the Student’s *t-*test, ANOVA and the Tukey *post hoc* test as indicated. Significance was set at *p* < 0.05.

## Results

### The Production of Putrescine Increases with Tyrosine Concentration

**FIGURE 3:**
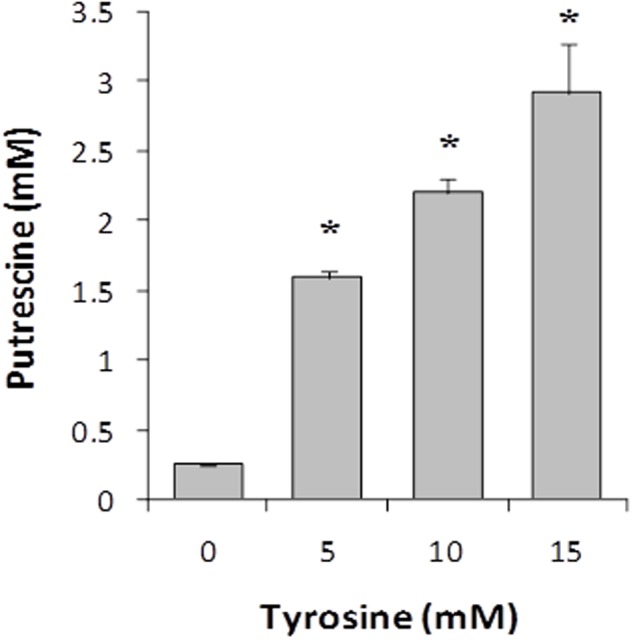
Effect of tyrosine concentration on putrescine accumulation in *E. faecalis* V583 cultures grown in GM17 in the presence of 5 mM agmatine. Asterisks indicate significant differences with respect to the previous condition (^∗^*p* < 0.01, ANOVA and Tukey *post hoc* tests).

The effect of the tyrosine (tyramine substrate) concentration of culture medium on the putrescine biosynthesis was studied by growing the wt strain in GM17 supplemented with 5 mM agmatine (putrescine substrate) and 0, 5, 10, and 15 mM tyrosine until the end of the exponential phase of growth (*t* = 7 h). Then, putrescine concentration was measured by UHPLC. As shown in **Figure 3**, putrescine biosynthesis correlated positively with the tyrosine concentration.

We also investigated the influence of agmatine concentration in tyramine biosynthesis. However, no significant differences were found (data not shown), suggesting no correlation between agmatine concentration and tyramine biosynthesis.

### Transcriptomic Studies Show an Induction of the Putrescine Biosynthesis Genes in the Presence of Tyrosine

**FIGURE 4:**
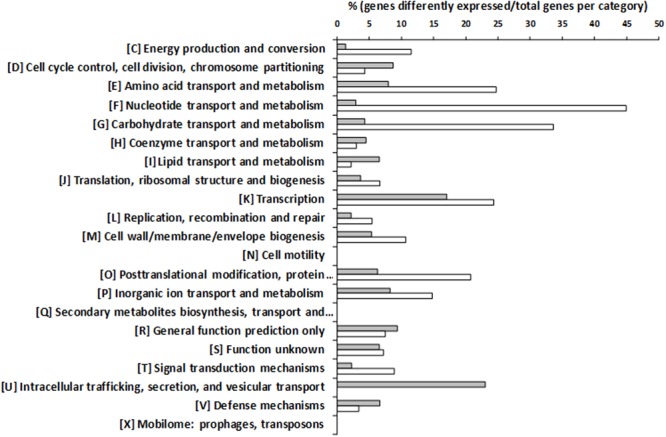
‘Clusters of Orthologous Groups’ (COG) classification of genes differentially expressed during the growth of *E. faecalis* in the presence of 15 mM tyrosine and 5 mM agmatine with respect to the same strain grown with 5 mM agmatine. Percentages of overexpressed genes are indicated by white bars and repressed genes by gray bars.

To seek for genes that could be involved in the observed increase of putrescine production in the presence of tyrosine, DNA microarrays were performed. The transcriptomic profile of the wt strain grown with or without tyrosine was compared. The expression of 394 genes was significantly different, 277 being induced in presence of tyrosine. In total, 12.4% of chromosomal genes showed an altered expression. Those genes with different expression were classified by the Clusters of Orthologous Groups (COG) categories of the National Centre for Biotechnology Information (NCBI) ^1^ (Supplementary Table S1) and their relevancies are shown in **Figure 4**. Most of the genes were assigned to the categories of nucleotides transport and metabolism, with the 48% of genes showing an altered expression, followed by carbohydrates (38%) and amino acids (33%) transport and metabolism categories. Furthermore, 41% of the genes assigned to transcription category showed a different expression.

As could be expected, the presence of tyrosine implied a repression of tyrosine biosynthesis, since most of the genes involved in tyrosine synthesis, including the shikimate pathway, were repressed (Supplementary Tables S1, S2 and Supplementary Figure S1).

Functional analysis of the differentially expressed genes made by GSEA, revealed the induction of pathways related with cell growth. Thus, it was observed an enhancement of purine and pyrimidine precursor supply routes related to DNA biosynthesis; an activation of aminosugars metabolism to provide precursors for cell wall biosynthesis; and an increment in the levels of aminoacyl-tRNA synthetases for protein biosynthesis (Supplementary Table S2 and Supplementary Figure S1).

Focusing on the genes involved in the BA biosynthesis, the expression of all the genes belonging to the *tdc* and *agdi* clusters was significantly different (**Table 1**). As expected, when *E. faecalis* was grown in the presence of tyrosine, the metabolic genes implicated in tyramine biosynthesis (*tdcA, tdcP*, and *nhac-2*) were induced. Furthermore, the genes responsible of putrescine biosynthesis (*aguB, aguD, aguA*, and *aguC*), were also induced in the presence of tyrosine.

**Table 1:** Expression of the *agdi* and *tdc* cluster genes in the transcriptome of *E. faecalis* V583 grown with or without 15 mM tyrosine.

	Gene	Locus	Description	Fold change	*p-*Value
***agdi cluster***	*aguR*	EF0731	Transcriptional regulator	2.48	6.81E-04
	*aguB*	EF0732	Putrescine carbamoyltransferase	8.05	5.24E-04
	*aguD*	EF0733	Agmatine/putrescine antiporter	6.88	4.18E-04
	*aguA*	EF0734	Agmatine deiminase	6.34	4.41E-04
	*aguC*	EF0735	Carbamate kinase	5.73	5.38E-04
***tdc cluster***	*tyrS*	EF0633	Tyrosyl-tRNAsynthetase	-8.73	2.41E-04
	*tdcA*	EF0634	Tyrosine decarboxylase	3.23	2.45E-04
	*tdcP*	EF0635	Tyrosine/tyramine exchanger	2.56	3.36E-04
	*nhac-2*	EF0636	Na^+^/H^+^ antiporter	2.21	6.04E-04

### RT-qPCR Results Confirm That Putrescine Biosynthesis Genes Are Induced by Tyrosine

To confirm the *agdi* cluster expression results obtained in the transcriptomic profile, we quantified by RT-qPCR the expression of *aguA* gene, as representative of the whole polycistronic *aguBDAC* mRNA. Total RNA was isolated from cultures of the wt strain grown with or without tyrosine, i.e., as they were cultivated for the microarray experiment.

**FIGURE 5:**
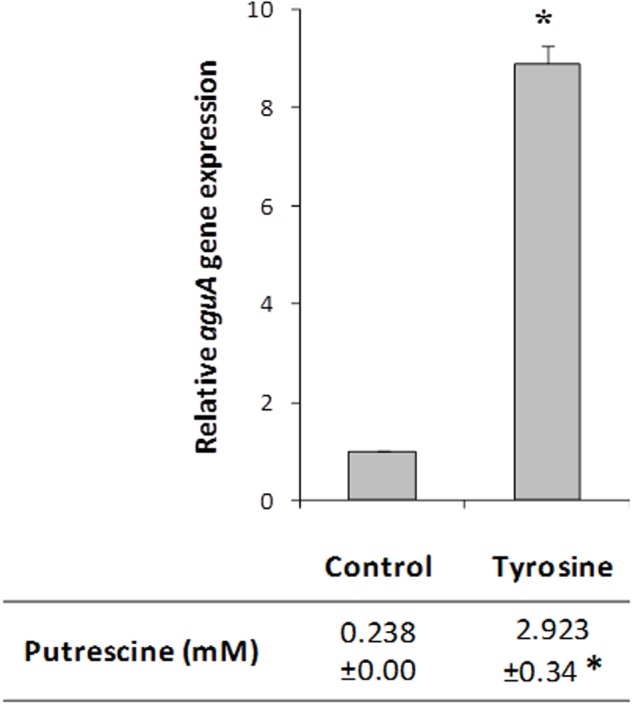
Effect of tyrosine on *aguA* expression (representing the whole *aguBDAC* operon) and putrescine production in *E. faecalis*. Cells were grown in GM17 supplemented with 5 mM agmatine (Control), 5 mM agmatine and 15 mM tyrosine. Gene expression was measured by RT-qPCR; putrescine accumulation was determined by UHPLC. The lowest expression level (Control) was used as the reference condition. Asterisks indicate significant differences with respect to the Control condition (^∗^*p* < 0.01, Student’s *t*-test).

The expression of *aguA* showed a significantly increase (ninefold) in the presence of tyrosine (**Figure 5**). In concordance with the previous results, putrescine biosynthesis reached about 10-fold concentrations when the amino acid tyrosine was added to the medium. These results support that tyrosine induces the putrescine biosynthesis genes.

### The *tdc* Cluster Is Involved in the Tyrosine Induction of Putrescine Biosynthesis

**FIGURE 6:**
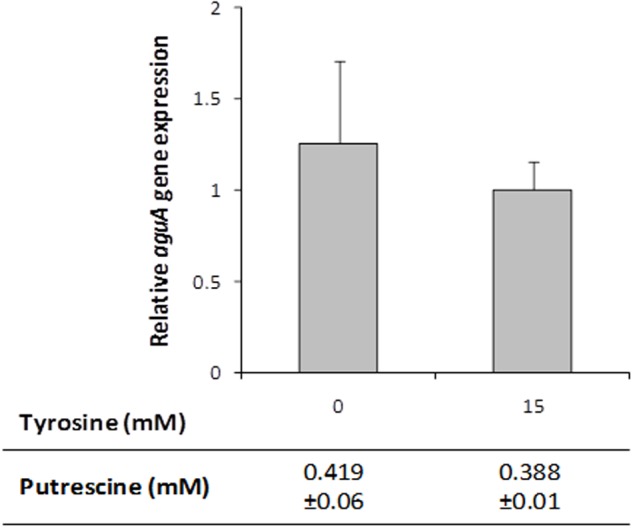
*aguA* expression and putrescine formation in *E. faecalis*Δ*tdc* cells grown in GM17 supplemented with 5 mM agmatine and in presence or absence of 15 mM tyrosine. Gene expression was measured by RT-qPCR and putrescine accumulation by UHPLC.

To test a potential role of the tyramine biosynthesis genes -the *tdc* cluster-, *aguA* expression and putrescine production were measured in cultures of the Δ*tdc* mutant strain grown in presence or absence of tyrosine. Unlike the wt strain results, no differences were observed in the *aguA* expression or putrescine production when tyrosine was added (**Figure 6**). This could be due to the fact that the product of the reaction, the tyramine – which is not produced in the Δ*tdc* mutant – was the inducer. To investigate it, the wt strain was grown with 5 mM agmatine and with and without 15 mM tyramine. No variation in *aguA* expression or putrescine production was observed when tyramine was present in the culture, indicating that it was not responsible of putrescine biosynthesis genes induction (data not shown).

### The Role of *aguR*-P*aguB* and *tdc* Cluster in the Induction by Tyrosine

**FIGURE 7:**
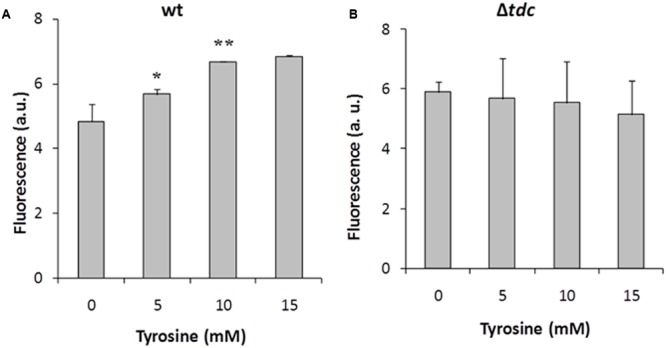
Induction of *gfp* in *E. faecalis* wt **(A)** and Δ*tdc* **(B)** cultures containing pAGEnt-GFP in the presence of 5 mM agmatine and under a range of tyrosine concentrations. Fluorescence was measured after 8 h of culture. Asterisks indicate significant differences with respect to the previous condition (^∗^*p* < 0.05, ^∗∗^*p* < 0.01, ANOVA and Tukey *post hoc* tests). a.u., arbitrary units.

To further study the tyrosine induction, we used the pAGEnt-GFP construct (**Figure 2**), in which the *gfp* gene is under the control of the *aguR*-P*aguB* cassette. The wt and Δ*tdc* strains harboring pAGEnt-GFP were grown for 8 h in medium supplemented with 5 mM agmatine and 0, 5, 10, or 15 mM tyrosine. Fluorescence measurements in the wt strain revealed that *gfp* expression correlated positively with the tyrosine concentration, reaching up to twofold units in presence of 10 mM tyrosine (**Figure 7A**). Conversely, no significantly differences were observed in the fluorescence signal of Δ*tdc* cultures (**Figure 7B**).

Hence, the protein AguR and the *aguB* promoter are both implicated in the regulation of the *aguBDAC* genes transcription mediated by the *tdc* cluster in the presence of tyrosine.

## Discussion

Previous studies in *E. faecalis* have shown that the genes responsible for tyrosine and putrescine biosynthesis are induced by their respective substrates, tyrosine and agmatine (Linares et al., 2014; Perez et al., 2015). Although the genetic regulation of different BA biosynthesis pathways in different microorganisms has been studied, possible co-regulation between different BA pathways within one bacterium has not been widely described. Since *E. faecalis* produces tyramine and putrescine, we examined the potential co-regulation among TDC and AGDI pathways, studying in principle whether the substrates have any effect on the other metabolic pathway. In fact, the higher the concentration of tyrosine, the greater the production of putrescine (**Figure 3**). Nevertheless, this effect was not reciprocal, since the production of tyramine remained constant independently of agmatine concentration.

A reciprocal association between tyramine and putrescine biosynthesis has been suggested in *Lactobacillus brevis* (Russo et al., 2012) showing an induction of both pathways when both precursors were present. In contrast, other authors observed the repression of the arginine deiminase pathway by histidine decarboxylase route in *Lactobacillus hilgardii* (Lamberti et al., 2011) and by glutamate decarboxylase pathway in *Lactococcus lactis* (Mazzoli et al., 2010) and *Lactobacillus reuteri* (Teixeira et al., 2014).

To deepen the genetic regulation, we further investigated the tyrosine effect on the transcriptome of *E. faecalis.* The *tdc* cluster transcription observed in the microarray experiment (**Table 1**) concurs with our previous results obtained by RT-qPCR when the effect of the substrate amino acid on *tdc* cluster expression was assessed (Perez et al., 2015). I.e., the catabolic genes *tdcA, tdcP*, and *nhac-2* – that encode a tyrosine decarboxylase, a tyrosine-tyramine antiporter and a Na^+^/H^+^ antiporter, respectively – are over-expressed by tyrosine, while the *tyrS* which encodes an aminoacyl-tRNA synthetase-like gene is repressed. This effect of tyrosine on *tdc* cluster expression has also been demonstrated in other *E. faecalis* strains by Bargossi et al. (2015, 2017).

Focusing on the putrescine biosynthesis genes, the global transcriptome analysis proved that tyrosine induces the catabolic genes of the *agdi* cluster (**Table 1**). The induction of the *agdi* catabolic operon by tyrosine was ascertained by RT-qPCR analysis of *aguA* expression, showing a rise of ninefold in the presence of tyrosine (**Figure 5**). Tyrosine induction of putrescine biosynthesis genes was not observed in the mutant strain Δ*tdc* (**Figure 7B**), which is unable to decarboxylate tyrosine and produce tyramine (Perez et al., 2015). Therefore, the product of the reaction, tyramine, could be the inducer. However, we found that tyramine had no effect on *aguA* expression or putrescine biosynthesis in the wt strain. Landete et al. (2007) also observed that tyramine does not affect putrescine production in other *E. faecalis* strains. In addition to the cluster *tdc* was necessary, the use of the *gfp* reporter gene established that tyrosine induction of the *agdi* cluster was performed throughout the *aguR*-P*aguB* cassette (**Figure 7A**). The phenotypic differences observed are smaller than those observed in transcriptome and RT-qPCR experiments. This could be explained by the lower sensitivity of the whole-cell fluorescence technique, and the contrast between the *agdi* cluster, which is located in the chromosome and the reporter gene located in a multicopy plasmid, which implies a greater background. The use of *gfp* also verified that the P*aguB* promoter is not induced by tyrosine in the mutant strain Δ*tdc* (**Figure 7B**).

Further analysis need to be done in order to investigate the mechanism of the induction exerted by tyrosine and the *tdc* cluster on the AGDI pathway. Since the transcriptional regulator AguR is a transmembrane protein (Linares et al., 2015), the antiporter TdcP or the tyrosine decarboxylase TdcA – which are membrane proteins (Pessione et al., 2009) – could interact with AguR enhancing its union to *aguB* promoter and inducing the expression of *aguBDAC*.

Tyrosine – besides being a BA precursor that induces the *tdc* transcription- could even work as an indicator of amino acids abundance for the bacterial cell and direct metabolism toward cell growth. In fact, functional analysis of the transcriptomic data indicated that a tyrosine excess inhibits its own biosynthesis route and activates pathways related to accumulation of precursors for DNA, glucolytic and pyruvate metabolism enzymes and cell wall biosynthesis. Moreover, an increment in the transcription of the genes encoding some aminoacyl-tRNA synthetases was also observed, suggesting the cell would be loading the corresponding tRNAs for protein biosynthesis. Supplementation with amino acids has been shown to act as a signal for growth in *Bacillus subtilis* by reducing the expression of genes related to amino acid biosynthesis and inducing genes involved in nucleotide metabolism (Mader et al., 2002; Pessione et al., 2009).

## Conclusion

Tyrosine, which is the substrate of tyramine biosynthesis, is also an inducer of putrescine production in *E. faecalis*, the main producer of both BA in cheese. Moreover, the presence of tyrosine would be received by the enterococci cells as a signal to growth, what would lead in an increment in the number of BA-producing cells. Therefore, tyrosine appears to be a relevant amino acid in cheese, in the sense that it would increase the risk of accumulating tyramine and putrescine, which are health threatening for consumers.

## Author Contributions

MP carried out most of experiments, analyzed data and drafted the manuscript, VL analyzed some of the data and help to write the manuscript; BdR and BR performed some experiments; AdJ, OK, JK, MM, and MF participated in study design and data interpretation; AdJ, OK, and JK supervised the arrays experiments; MA provided the general concept, participated in study design and data interpretation, and supervised the work and the manuscript. All authors contributed to the discussion of the research and approved the final manuscript.

## Conflict of Interest Statement

The authors declare that the research was conducted in the absence of any commercial or financial relationships that could be construed as a potential conflict of interest.

## Footnotes

1 www.ncbi.nlm.nih.gov/COG/
