# Supplementary material for: The Relationship among Tyrosine Decarboxylase and Agmatine Deiminase Pathways in Enterococcus faecalis
Source: Front Microbiol. 2017 Nov 1;8:2107. doi: 10.3389/fmicb.2017.02107 (PMC5672081; doi:10.3389/fmicb.2017.02107)
Supplement: Supplementary file 1 [file Table_1.DOCX]

Table S1. Genes differentially expressed in *E. faecalis* V583 grown with 15 mM tyrosine and 5 mM agmatine, and *E. faecalis* V583 grown with 5 mM agmatine. Genes with an at least 2-fold difference in expression, associated with a *p* value of 0.001 are shown. Genes were classified in terms of 'Clusters of Orthologous Groups' (COG) categories. ^a^Description according to the NCBI annotation. ^b^Function and subsystem of the Rapid Annotation using Subsystem Technology (RAST) annotation of the National Microbial Pathogen Data Resource (NMPDR). Genes belonging to *tdc* and *agdi* clusters are shaded.

| **LOCUS** | **GENE** | **FOLD** | ***p*-VALUE** | **DESCRIPTION^a^** | **FUNCTION^b^** | **SUBSYSTEM^b^** |
| --- | --- | --- | --- | --- | --- | --- |
| **[C] Energy production and conversion** | | | | | | |
| EF0402 | *nhaC-1* | -10.99 | 4,94E-05 | Na+/H+ antiporter | Predicted tyrosine transporter, NhaC family | - none - |
| EF0636 | *nhaC-2* | 2.21 | 6,04E-04 | Na+/H+ antiporter | Predicted tyrosine transporter, NhaC family | - none - |
| EF0895 | *-* | 2.27 | 5,36E-04 | glycerol dehydrogenase | Glycerol dehydrogenase (EC 1,1,1,6) | Respiratory dehydrogenases 1 |
| EF0900 | *adhE* | 6.43 | 9,49E-05 | bifunctional acetaldehyde-CoA/alcohol dehydrogenase | Alcohol dehydrogenase (EC 1,1,1,1); Acetaldehyde dehydrogenase (EC 1,2,1,10) | 5-FCL-like protein; fermentations: lactate; fermentations: mixed acid; fermentations: mixed acid; glycerolipid and glycerophospholipid metabolism in bacteria; pyruvate metabolism II: acetyl-CoA, acetogenesis from pyruvate |
| EF1108 | *-* | 4.19 | 3,24E-04 | oxidoreductase | Predicted L-lactate dehydrogenase, Fe-S oxidoreductase subunit YkgE | Lactate utilization |
| EF1109 | *-* | 3.78 | 7,93E-04 | iron-sulphur cluster binding protein | Predicted L-lactate dehydrogenase, Iron-sulphur cluster-binding subunit YkgF | L-rhamnose utilization; lactate utilization |
| EF1207 | *-* | 2.93 | 2,26E-04 | CCS family citrate carrier protein | Malate Na(+) symporter | Pyruvate metabolism I: anaplerotic reactions, PEP |
| EF1358 | *gldA* | 14.81 | 2,40E-05 | glycerol dehydrogenase | Glycerol dehydrogenase (EC 1,1,1,6) | Respiratory dehydrogenases 1 |
| EF2570 | *-* | 2.87 | 2,20E-04 | aldehyde oxidoreductase | Xanthine dehydrogenase iron-sulphur subunit (EC 1,17,1,4) / Xanthine dehydrogenase, molybdenum binding subunit (EC 1,17,1,4) | Purine Utilization; purine utilization; xanthine dehydrogenase subunits; xanthine dehydrogenase subunits |
| EF3036 | *-* | 2.43 | 3,16E-04 | thioredoxin family protein | FIG009688: Thioredoxin | - none - |
| **[D] Cell cycle control, cell division, chromosome partitioning** | | | | | | |
| EF1761 | *ftsE* | -2.7 | 5,39E-04 | cell division ATP-binding protein FtsE | Cell division transporter, ATP-binding protein FtsE (TC 3,A,5,1,1) | Bacterial cell division; CBSS-393121,3,peg,2760 |
| EF2533 | *-* | 3.44 | 1,62E-04 | FtsK/SpoIIIE family protein | FIG00630048: hypothetical protein | - none - |
| EF3311 | *gidA* | -3.26 | 2,94E-04 | tRNA uridine 5-carboxymethylaminomethyl modification protein GidA | tRNA uridine 5-carboxymethylaminomethyl modification enzyme GidA | RNA modification and chromosome partitioning cluster; mnm5U34 biosynthesis bacteria; tRNA modification Bacteria |
| **[E] Amino acid transport and metabolism** | | | | | | |
| EF0063 | *-* | 2.47 | 6,53E-04 | pheromone binding protein | Oligopeptide ABC transporter, periplasmic oligopeptide-binding protein OppA (TC 3,A,1,5,1) | ABC transporter oligopeptide (TC 3,A,1,5,1); sex pheromones in *Enterococcus faecalis* and other Firmicutes |
| EF0108 | *-* | 4.9 | 4,43E-05 | C4-dicarboxylate transporter | Arginine/ornithine antiporter ArcD | Arginine deiminase pathway; arginine and ornithine degradation; polyamine metabolism |
| EF0247 | *-* | 2.78 | 3,75E-04 | amino acid ABC transporter amino acid-binding/permease | Amino acid ABC transporter, glutamine-binding protein/permease protein | - none - |
| EF0302 | *pepC* | 2.37 | 3,84E-04 | aminopeptidase C | Aminopeptidase C (EC 3,4,22,40) | Protein degradation |
| EF0634 | *tdcA* | 3.23 | 2,45E-04 | decarboxylase | decarboxylase, putative | - none - |
| EF0635 | *tdcP* | 2.56 | 3,36E-04 | amino acid permease | amino acid permease family protein | - none - |
| EF0732 | *aguB* | 8.05 | 5,24E-04 | putrescine carbamoyltransferase | Putrescine carbamoyltransferase (EC 2,1,3,6) | Polyamine metabolism |
| EF0733 | *aguD* | 6.88 | 4,18E-04 | amino acid permease | Agmatine/putrescine antiporter, associated with agmatine catabolism | Polyamine metabolism |
| EF0734 | *aguA* | 6.34 | 4,41E-04 | agmatine deiminase | Agmatine deiminase (EC 3,5,3,12) | Arginine and ornithine degradation; polyamine metabolism |
| EF0735 | *aguC* | 5.73 | 5,38E-04 | carbamate kinase | Carbamate kinase (EC 2,7,2,2) | Arginine deiminase pathway; arginine and ornithine degradation; polyamine metabolism |
| EF0804 | *-* | 2.64 | 2,05E-04 | amino acid ABC transporter amino acid-binding protein | Amino acid ABC transporter, amino acid-binding protein | - none - |
| EF0805 | *-* | 2.59 | 3,14E-04 | amino acid ABC transporter ATP-binding protein | ABC transporter | - none - |
| EF0892 | *-* | 2.03 | 8,16E-04 | amino acid ABC transporter ATP-binding protein | amino acid ABC transporter, ATP-binding protein | - none - |
| EF0907 | *-* | 5.34 | 1,46E-04 | peptide ABC transporter peptide-binding protein | Oligopeptide ABC transporter, periplasmic oligopeptide-binding protein OppA (TC 3,A,1,5,1) | ABC transporter oligopeptide (TC 3,A,1,5,1); sex pheromones in *Enterococcus faecalis* and other Firmicutes |
| EF1117 | *-* | 5.28 | 6,63E-05 | amino acid ABC transporter permease | Glutamate transport membrane-spanning protein | - none - |
| EF1118 | *-* | 5.85 | 3,18E-05 | amino acid ABC transporter permease | Glutamate transport permease protein | - none - |
| EF1119 | *-* | 8.28 | 2,10E-05 | amino acid ABC transporter amino acid-binding protein | Glutamine ABC transporter, periplasmic glutamine-binding protein (TC 3,A,1,3,2) | - none - |
| EF1120 | *-* | 5.65 | 2,30E-05 | amino acid ABC transporter ATP-binding protein | amino acid ABC transporter ATP-binding protein | - none - |
| EF1218 | *-* | 6.53 | 5,75E-05 | spermidine/putrescine ABC transporter permease | Spermidine Putrescine ABC transporter permease component PotB (TC 3,A,1,11,1) | Polyamine metabolism |
| EF1219 | *-* | 30.25 | 9,88E-06 | spermidine/putrescine ABC transporter permease | Spermidine Putrescine ABC transporter permease component PotC (TC_3,A,1,11,1) | Polyamine metabolism |
| EF1220 | *-* | 37.45 | 9,87E-06 | spermidine/putrescine ABC transporter ATP-binding protein | Putrescine transport ATP-binding protein PotA (TC 3,A,1,11,1) | Polyamine metabolism |
| EF1221 | *-* | 20.93 | 2,39E-05 | spermidine/putrescine ABC transporter spermidine/putrescine-binding protein | ABC transporter, periplasmic spermidine putrescine-binding protein PotD (TC 3,A,1,11,1) | Polyamine metabolism |
| EF1415 | *gdhA* | 2.13 | 5,11E-04 | glutamate dehydrogenase | NADP-specific glutamate dehydrogenase (EC 1,4,1,4) | Arginine and ornithine degradation; glutamate dehydrogenases; glutamine, glutamate, aspartate and asparagine biosynthesis; proline synthesis |
| EF1561 | *aroE* | -9.18 | 1,22E-05 | shikimate 5-dehydrogenase | Shikimate/quinate 5-dehydrogenase I beta (EC 1,1,1,282) | Chorismate synthesis; common pathway for synthesis of aromatic compounds (DAHP synthase to chorismate); quinate degradation |
| EF1562 | *-* | -9.03 | 1,57E-05 | 3-deoxy-7-phosphoheptulonate synthase | 2-keto-3-deoxy-D-arabino-heptulosonate-7-phosphate synthase I beta (EC 2,5,1,54) | Chorismate synthesis; common pathway for synthesis of aromatic compounds (DAHP synthase to chorismate) |
| EF1563 | *aroB* | -10.46 | 1,35E-05 | 3-dehydroquinate synthase | 3-dehydroquinate synthase (EC 4,2,3,4) | Chorismate synthesis; common pathway for synthesis of aromatic compounds (DAHP synthase to chorismate) |
| EF1564 | *aroC* | -9.04 | 2,21E-05 | chorismate synthase | Chorismate synthase (EC 4,2,3,5) | Chorismate synthesis; common pathway for synthesis of aromatic compounds (DAHP synthase to chorismate) |
| EF1565 | *-* | -9.06 | 1,12E-05 | prephenate dehydrogenase | Prephenate dehydrogenase (EC 1,3,1,12) | Chorismate synthesis; phenylalanine and tyrosine branches from chorismate |
| EF1566 | *aroA* | -10.25 | 1,29E-05 | 3-phosphoshikimate 1-carboxyvinyltransferase | 5-Enolpyruvylshikimate-3-phosphate synthase (EC 2,5,1,19) | Chorismate synthesis; common pathway for synthesis of aromatic compounds (DAHP synthase to chorismate) |
| EF1567 | *aroK* | -8.92 | 1,43E-05 | shikimate kinase | Shikimate kinase I (EC 2,7,1,71) | Chorismate synthesis; common pathway for synthesis of aromatic compounds (DAHP synthase to chorismate) |
| EF1568 | *-* | -8.78 | 2,79E-05 | prephenate dehydratase | Prephenate dehydratase (EC 4,2,1,51) | Chorismate synthesis; phenylalanine and tyrosine branches from chorismate |
| EF2652 | *-* | 2.65 | 2,54E-04 | spermidine/putrescine ABC transporter ATP-binding protein | Putrescine transport ATP-binding protein PotA (TC 3,A,1,11,1) | Polyamine metabolism |
| EF3272 | *-* | 2.03 | 6,96E-04 | zinc-binding Cro/CI family transcriptional regulator | zinc-binding transcriptional regulator, Cro/CI family | - none - |
| **[F] Nucleotide transport and metabolism** | | | | | | |
| EF0014 | *purA* | 5.51 | 4,53E-05 | adenylosuccinate synthetase | Adenylosuccinate synthetase (EC 6,3,4,4) | Purine conversions |
| EF0173 | *pyn* | 4.31 | 5,76E-05 | pyrimidine-nucleoside phosphorylase | Pyrimidine-nucleoside phosphorylase (EC 2,4,2,2) | Deoxyribose and deoxynucleoside catabolism; pyrimidine conversions |
| EF0174 | *deoC* | 3.73 | 7,86E-05 | deoxyribose-phosphate aldolase | Deoxyribose-phosphate aldolase (EC 4,1,2,4) | Deoxyribose and deoxynucleoside catabolism |
| EF0175 | *cdd* | 4.3 | 5,90E-05 | cytidine deaminase | Cytidine deaminase (EC 3,5,4,5) | Murein hydrolase regulation and cell death; pyrimidine conversions; tRNA modification bacteria |
| EF0176 | *-* | 3.9 | 8,09E-05 | hypothetical protein | Predicted nucleoside ABC transporter, substrate-binding component | D-ribose utilization; deoxyribose and deoxynucleoside catabolism |
| EF0177 | *-* | 3.83 | 8,37E-05 | hypothetical protein | Predicted nucleoside ABC transporter, substrate-binding component | D-ribose utilization; deoxyribose and deoxynucleoside catabolism |
| EF0178 | *-* | 2.53 | 3,44E-04 | ABC transporter ATP-binding protein | Predicted nucleoside ABC transporter, ATP-binding component | D-ribose utilization; deoxyribose and deoxynucleoside catabolism |
| EF0180 | *-* | 2.17 | 7,60E-04 | ABC transporter permease | Unspecified monosaccharide ABC transport system, permease component 2 | - none - |
| EF0186 | *deoD-1* | 2.12 | 5,09E-04 | purine nucleoside phosphorylase | purine nucleoside phosphorylase( EC:2,4,2,1 ) | - none - |
| EF1036 | *-* | 2.14 | 6,64E-04 | nucleoside diphosphate kinase | Nucleoside diphosphate kinase (EC 2,7,4,6) | Purine conversions; pyrimidine conversions |
| EF1075 | *-* | 3.22 | 3,23E-04 | acetyltransferase | acetyltransferase, GNAT family | - none - |
| EF1076 | *-* | 3.85 | 6,39E-05 | streptomycin 3''-adenylyltransferase | streptomycin 3''-adenylyltransferase, putative | - none - |
| EF1147 | *pyrG* | 2.47 | 5,81E-04 | CTP synthetase | CTP synthase (EC 6,3,4,2) | Ribosome post-transcriptional modification and chromosomal segregation cluster; pyrimidine conversions |
| EF1222 | *ade* | 34.01 | 1,41E-05 | adenine deaminase | Adenine deaminase (EC 3,5,4,2) | Purine conversions |
| EF1223 | *-* | 23.16 | 1,81E-05 | chlorohydrolase | Cytosine deaminase | - none - |
| EF1547 | *cmk* | 2.41 | 3,19E-04 | cytidylate kinase | Cytidylate kinase (EC 2,7,4,14) | Ribosome post-transcriptional modification and chromosomal segregation cluster; pyrimidine conversions |
| EF1777 | *purD* | 5.6 | 3,82E-05 | phosphoribosylamine--glycine ligase | Phosphoribosylamine--glycine ligase (EC 6,3,4,13) | De novo purine biosynthesis |
| EF1778 | *purH* | 10.37 | 1,75E-05 | phosphoribosylaminoimidazolecarboxamide formyltransferase/IMP cyclohydrolase | IMP cyclohydrolase (EC 3,5,4,10) / Phosphoribosylaminoimidazolecarboxamide formyltransferase (EC 2,1,2,3) | 5-FCL-like protein; CBSS-366602,3,peg,5141; CBSS-366602,3,peg,5141; de novo purine biosynthesis; de novo purine biosynthesis |
| EF1779 | *purN* | 14.48 | 3,70E-05 | phosphoribosylglycinamide formyltransferase | Phosphoribosylglycinamide formyltransferase (EC 2,1,2,2) | 5-FCL-like protein; de novo purine biosynthesis |
| EF1780 | *purM* | 5.19 | 1,37E-04 | phosphoribosylaminoimidazole synthetase | Phosphoribosylformylglycinamidine cyclo-ligase (EC 6,3,3,1) | De novo purine biosynthesis |
| EF1781 | *purF* | 11.03 | 2,49E-05 | amidophosphoribosyltransferase | Amidophosphoribosyltransferase (EC 2,4,2,14) | Colicin V and bacteriocin production cluster; de novo purine biosynthesis |
| EF1782 | *purL* | 10.44 | 2,70E-05 | phosphoribosylformylglycinamidine synthase II | Phosphoribosylformylglycinamidine synthase, synthetase subunit (EC 6,3,5,3) | De novo purine biosynthesis |
| EF1783 | *purQ* | 16.37 | 1,54E-05 | phosphoribosylformylglycinamidine synthetase I | Phosphoribosylformylglycinamidine synthase, glutamine amidotransferase subunit (EC 6,3,5,3) | De novo purine biosynthesis |
| EF1784 | *purS* | 12.37 | 1,30E-05 | phosphoribosylformylglycinamidine synthase PurS | Phosphoribosylformylglycinamidine synthase, PurS subunit (EC 6,3,5,3) | De novo purine biosynthesis |
| EF1785 | *purC* | 16.24 | 1,34E-05 | phosphoribosylaminoimidazole-succinocarboxamide synthase | Phosphoribosylaminoimidazole-succinocarboxamide synthase (EC 6,3,2,6) | De novo purine biosynthesis |
| EF1786 | *purK-1* | 12.18 | 1,28E-05 | phosphoribosylaminoimidazole carboxylase ATPase subunit | Phosphoribosylaminoimidazole carboxylase ATPase subunit (EC 4,1,1,21) | De novo purine biosynthesis |
| EF1787 | *purE* | 3.28 | 1,98E-04 | phosphoribosylaminoimidazole carboxylase catalytic subunit | Phosphoribosylaminoimidazole carboxylase catalytic subunit (EC 4,1,1,21) | De novo purine biosynthesis |
| EF1921 | *-* | 5.08 | 4,83E-05 | ribonucleoside hydrolase RihC | Inosine-uridine preferring nucleoside hydrolase (EC 3,2,2,1) | Purine conversions; queuosine-archaeosine biosynthesis |
| EF2073 | *prsA-1* | -2.13 | 6,40E-04 | ribose-phosphate pyrophosphokinase | Ribose-phosphate pyrophosphokinase (EC 2,7,6,1) | A Gammaproteobacteria cluster relating to translation; de novo purine Biosynthesis; pentose phosphate pathway; transcription repair cluster |
| EF2429 | *guaC* | 13.68 | 1,64E-05 | guanosine 5'-monophosphate oxidoreductase | GMP reductase (EC 1,7,1,7) | Purine conversions |
| EF2430 | *-* | 15.42 | 1,37E-05 | xanthine/uracil permease | xanthine/uracil permease family protein | - none - |
| EF2431 | *-* | 12.71 | 2,25E-05 | chlorohydrolase | Guanine deaminase (EC 3,5,4,3) | Purine Utilization; purine conversions |
| EF3127 | *gmk* | -3.11 | 1,36E-04 | guanylate kinase | Guanylate kinase (EC 2,7,4,8) | CBSS-323097,3,peg,2594; purine conversions |
| **[G] Carbohydrate transport and metabolism** | | | | | | |
| EF0020 | *-* | 3.36 | 3,63E-04 | PTS system mannose-specfic transporter subunit IIAB | PTS system, mannose-specific IIB component (EC 2,7,1,69) / PTS system, mannose-specific IIA component (EC 2,7,1,69) | Mannose metabolism; mannose metabolism; sialic acid metabolism; sialic acid metabolism |
| EF0021 | *-* | 3.77 | 4,56E-04 | PTS system mannose-specfic transporter subunit IIC | PTS system, mannose-specific IIC component (EC 2,7,1,69) | Mannose metabolism; sialic acid metabolism |
| EF0022 | *-* | 3.93 | 1,33E-04 | PTS system mannose-specfic transporter subunit IID | PTS system, mannose-specific IID component (EC 2,7,1,69) | Mannose metabolism; sialic acid metabolism |
| EF0071 | *-* | 3.7 | 2,60E-04 | lipoprotein | Putative isomerase | - none - |
| EF0082 | *-* | -3.39 | 5,17E-04 | major facilitator family transporter | transporter | - none - |
| EF0424 | *-* | 2.11 | 9,34E-04 | 2-dehydro-3-deoxygluconokinase | 2-dehydro-3-deoxygluconate kinase (EC 2,7,1,45) | D-gluconate and ketogluconates metabolism; Entner-Doudoroff pathway |
| EF0677 | *-* | 4.95 | 9,61E-05 | phosphoglucomutase/phosphomannomutase | Phosphoglucosamine mutase (EC 5,4,2,10) | Sialic acid metabolism; UDP-N-acetylmuramate from fructose-6-phosphate biosynthesis |
| EF0717 | *-* | 3.59 | 7,24E-05 | PTS system fructose-specific transporter subunit IIABC | PTS system, fructose-specific IIA component (EC 2,7,1,69) / PTS system, fructose-specific IIB component (EC 2,7,1,69) / PTS system, fructose-specific IIC component (EC 2,7,1,69) | Fructose utilization; fructose utilization; fructose utilization |
| EF0718 | *fruK-2* | 12.23 | 3,05E-05 | 1-phosphofructokinase | 1-phosphofructokinase( EC:2,7,1,56 ) | - none - |
| EF0928 | *-* | -2.34 | 4,94E-04 | glucose uptake protein | glucose uptake protein | - none - |
| EF0938 | *-* | 5 | 9,17E-05 | sugar ABC transporter ATP-binding protein | Multiple sugar ABC transporter, ATP-binding protein | Fructooligosaccharides(FOS) and raffinose utilization; maltose and maltodextrin utilization |
| EF1012 | *-* | 6.28 | 7,75E-05 | PTS system transporter subunit IIB | PTS system, cellobiose-specific IIB component (EC 2,7,1,69) | Beta-glucoside metabolism |
| EF1017 | *-* | 5.79 | 5,43E-05 | PTS system transporter subunit IIB | PTS system, cellobiose-specific IIB component (EC 2,7,1,69) | Beta-glucoside metabolism |
| EF1018 | *-* | 19.06 | 1,29E-04 | PTS system transporter subunit IIA | PTS system, cellobiose-specific IIA component (EC 2,7,1,69) | Beta-glucoside metabolism |
| EF1019 | *-* | 6.2 | 3,29E-04 | PTS system transporter subunit IIC | PTS system, cellobiose-specific IIC component (EC 2,7,1,69) | Beta-glucoside metabolism |
| EF1068 | *galM* | 2.2 | 4,57E-04 | aldose 1-epimerase | Aldose 1-epimerase (EC 5,1,3,3) | Lactose and galactose uptake and utilization; maltose and maltodextrin utilization |
| EF1069 | *galK* | 4.49 | 5,35E-05 | galactokinase | Galactokinase (EC 2,7,1,6) | Lactose and galactose uptake and utilization |
| EF1159 | *-* | 4.19 | 2,42E-04 | PTS system cellobiose-specific transporter subunit IIB | PTS system, cellobiose-specific component BII | - none - |
| EF1192 | *-* | -2.94 | 1,30E-04 | aquaporin Z | Aquaporin Z | Osmoregulation |
| EF1243 | *-* | 6.9 | 3,82E-05 | glycosyl hydrolase | Beta-glucosidase (EC 3,2,1,21); 6-phospho-beta-glucosidase (EC 3,2,1,86) | Beta-glucoside metabolism; beta-glucoside metabolism; Fructooligosaccharides(FOS) and raffinose utilization |
| EF1345 | *-* | 9.31 | 2,43E-05 | sugar ABC transporter sugar-binding protein | Maltose/maltodextrin ABC transporter, substrate binding periplasmic protein MalE | Alpha-amylase locus in *Streptocococcus;* bacterial chemotaxis; maltose and maltodextrin utilization |
| EF1360 | *-* | 5.46 | 1,78E-04 | dihydroxyacetone kinase | Phosphoenolpyruvate-dihydroxyacetone phosphotransferase (EC 2,7,1,121), dihydroxyacetone binding subunit DhaK | Dihydroxyacetone kinases |
| EF1361 | *-* | 4.31 | 2,60E-04 | dihydroxyacetone kinase | Phosphoenolpyruvate-dihydroxyacetone phosphotransferase (EC 2,7,1,121), ADP-binding subunit DhaL | Dihydroxyacetone kinases |
| EF1503 | *-* | 4.4 | 1,23E-04 | fructose-1,6-bisphosphatase | Fructose-1,6-bisphosphatase, Bacillus type (EC 3,1,3,11) | Glycolysis and gluconeogenesis |
| EF1526 | *gap-1* | 2.44 | 7,83E-04 | glyceraldehyde 3-phosphate dehydrogenase | NAD-dependent glyceraldehyde-3-phosphate dehydrogenase (EC 1,2,1,12) | Entner-Doudoroff pathway; glycolysis and gluconeogenesis; redox-dependent regulation of nucleus processes |
| EF1603 | *scrB-1* | -2.54 | 3,56E-04 | sucrose-6-phosphate dehydrogenase | Sucrose-6-phosphate hydrolase (EC 3,2,1,26) | Fructooligosaccharides(FOS) and raffinose utilization; sucrose utilization |
| EF1179 | *cscK* | 7.3 | 2,09E-05 | fructokinase | Fructokinase (EC 2,7,1,4) | Fructose utilization; mannitol utilization; sucrose utilization |
| EF1807 | *lacD-2* | 2.32 | 3,80E-04 | tagatose 1,6-diphosphate aldolase | Tagatose 1,6-diphosphate aldolase (EC 4,1,2,40) | Lactose and galactose uptake and utilization |
| EF1836 | *-* | 2.33 | 4,36E-04 | PTS system transporter subunit IIA | PTS system, galactose-specific IIA component (EC 2,7,1,69) | Lactose and galactose uptake and utilization |
| EF1837 | *-* | 2.36 | 2,75E-04 | PTS system transporter subunit IIB | PTS system, galactose-specific IIB component (EC 2,7,1,69) | Lactose and galactose uptake and utilization |
| EF1912 | *-* | 5.47 | 1,07E-04 | ROK family protein | N-acetylmannosamine kinase (EC 2,7,1,60) | Sialic Acid Metabolism |
| EF2213 | *-* | 9.78 | 1,31E-05 | PTS system transporter subunit IIBC | PTS system, trehalose-specific IIB component (EC 2,7,1,69) / PTS system, trehalose-specific IIC component (EC 2,7,1,69) | Trehalose uptake and utilization; trehalose uptake and utilization |
| EF2221 | *-* | 3.25 | 2,37E-04 | ABC transporter substrate-binding protein | Multiple sugar ABC transporter, substrate-binding protein | Fructooligosaccharides(FOS) and raffinose utilization |
| EF2222 | *-* | 3.62 | 3,62E-04 | ABC transporter permease | Multiple sugar ABC transporter, membrane-spanning permease protein MsmG | Fructooligosaccharides(FOS) and Raffinose Utilization |
| EF2223 | *-* | 22.14 | 1,11E-05 | ABC transporter permease | Multiple sugar ABC transporter, membrane-spanning permease protein MsmF | Fructooligosaccharides(FOS) and raffinose utilization |
| EF2425 | *-* | 2.25 | 3,13E-04 | phosphoglucomutase/phosphomannomutase | Phosphoglucosamine mutase (EC 5,4,2,10) / Phosphomannomutase (EC 5,4,2,8) | Mannose metabolism; sialic acid metabolism; UDP-N-acetylmuramate from fructose-6-phosphate biosynthesis |
| EF2664 | *-* | -2.48 | 6,92E-04 | phosphoglycerate mutase | Phosphoglycerate mutase family 5 | Phosphoglycerate mutase protein family |
| EF2863 | *-* | 2.03 | 8,74E-04 | endo-beta-N-acetylglucosaminidase | secreted endo-beta-N-acetylglucosaminidase | - none - |
| EF2960 | *-* | 5.75 | 7,13E-05 | D-ribose pyranase | Ribose ABC transport system, high affinity permease RbsD (TC 3,A,1,2,1) | D-ribose utilization |
| EF2961 | *rbsK* | 10.74 | 8,14E-05 | ribokinase | Ribokinase (EC 2,7,1,15) | D-ribose utilization; deoxyribose and deoxynucleoside catabolism |
| EF2965 | *-* | 9.21 | 4,76E-05 | hypothetical protein | FIG00629163: hypothetical protein | - none - |
| EF3031 | *-* | 2.64 | 1,49E-04 | PTS system transporter subunit IIB | PTS system, mannose-specific IIB component (EC 2,7,1,69) | Mannose metabolism; sialic acid metabolism |
| EF3037 | *pepA* | 2.46 | 2,35E-04 | glutamyl-aminopeptidase | glutamyl-aminopeptidase( EC:3,4,11,7 ) | - none - |
| EF3142 | *-* | 6.17 | 3,25E-05 | 6-phosphogluconate dehydrogenase | 6-phosphogluconate dehydrogenase, decarboxylating (EC 1,1,1,44) | D-gluconate and ketogluconates metabolism; pentose phosphate pathway |
| **[H] Coenzyme transport and metabolism** | | | | | | |
| EF1225 | *-* | -6.02 | 2,52E-05 | thiamin biosynthesis ApbE | Thiamin biosynthesis lipoprotein ApbE | Iron-sulphur cluster assembly |
| EF1226 | *-* | -4.32 | 1,06E-04 | oxidoreductase | Fumarate reductase, flavoprotein subunit precursor (EC 1,3,99,1) | - none - |
| EF1227 | *-* | -3.37 | 2,47E-04 | hypothetical protein | Fumarate reductase, flavoprotein subunit precursor (EC 1,3,99,1) | - none - |
| EF2445 | *-* | 2.51 | 5,69E-04 | 2-dehydropantoate 2-reductase | 2-dehydropantoate 2-reductase (EC 1,1,1,169) | Coenzyme A biosynthesis |
| EF2776 | *thiE* | 2.35 | 8,82E-04 | thiamine-phosphate pyrophosphorylase | Thiamin-phosphate pyrophosphorylase (EC 2,5,1,3) | 5-FCL-like protein; thiamin biosynthesis |
| **[I] Lipid transport and metabolism** | | | | | | |
| EF0631 | *-* | -2.44 | 8,28E-04 | cardiolipin synthetase | Cardiolipin synthetase (EC 2,7,8,-) | Cardiolipin synthesis; glycerolipid and glycerophospholipid metabolism in bacteria |
| EF0848 | *acpS* | -2.69 | 5,91E-04 | holo-ACP synthase | Holo-[acyl-carrier protein] synthase (EC 2,7,8,7) | CBSS-176299,4,peg,1292; Fatty acid biosynthesis FASII |
| EF2601 | *-* | 2.03 | 6,45E-04 | acyl carrier protein | FMN-dependent NADH-azoreductase | - none - |
| EF2885 | *fabH* | -2.32 | 5,84E-04 | 3-oxoacyl-ACP synthase | 3-oxoacyl-[acyl-carrier-protein] synthase, KASIII (EC 2,3,1,41) | Fatty acid biosynthesis FASII |
| **[J] Translation, ribosomal structure and biogenesis** | | | | | | |
| EF0256 | *pth* | 2.75 | 8,07E-04 | peptidyl-tRNA hydrolase | Peptidyl-tRNA hydrolase (EC 3,1,1,29) | Cell division-ribosomal stress proteins cluster; sporulation-associated proteins with broader functions; transcription repair cluster; translation termination factors bacterial |
| EF0259 | *-* | 3.03 | 3,68E-04 | S4 RNA-binding domain-containing protein | Ribosome-associated heat shock protein implicated in the recycling of the 50S subunit (S4 paralog) | Cell division-ribosomal stress proteins cluster; DNA replication cluster 1; heat shock dnaK gene cluster extended |
| EF0633 | *tryS-1* | -8.73 | 2,41E-04 | tyrosyl-tRNA synthetase | Tyrosyl-tRNA synthetase (EC 6,1,1,1) | tRNA aminoacylation, Tyr |
| EF0678 | *-* | 4.43 | 7,86E-05 | acetyltransferase | Ribosomal-protein-S5p-alanine acetyltransferase | Ribosomal protein S5p acylation; Ribosome biogenesis bacterial |
| EF0697 | *-* | 2.36 | 4,18E-04 | hypothetical protein | Cysteinyl-tRNA synthetase related protein | CBSS-261594,1,peg,788 |
| EF0968 | *rplU* | -2.31 | 8,45E-04 | 50S ribosomal protein L21 | LSU ribosomal protein L21p | CBSS-176279,3,peg,868; ribosome LSU bacterial |
| EF0969 | *-* | -2.12 | 7,32E-04 | hypothetical protein | FIG139598: Potential ribosomal protein | CBSS-176279,3,peg,868 |
| EF1171 | *rpmE* | -3.39 | 2,20E-04 | 50S ribosomal protein L31 | LSU ribosomal protein L31p @ LSU ribosomal protein L31p, zinc-independent | Ribosome LSU bacterial; ribosome LSU bacterial |
| EF1970 | *aspS* | 2.99 | 4,27E-04 | aspartyl-tRNA synthetase | Aspartyl-tRNA synthetase (EC 6,1,1,12) | tRNA aminoacylation, Asp and Asn |
| EF1971 | *hisS* | 2.6 | 2,81E-04 | histidyl-tRNA synthetase | Histidyl-tRNA synthetase (EC 6,1,1,21) | tRNA aminoacylation, His |
| EF2070 | *mnmA* | -2.02 | 7,70E-04 | tRNA-specific 2-thiouridylase MnmA | tRNA-specific 2-thiouridylase MnmA | RNA methylation |
| EF2143 | *-* | 2.05 | 8,06E-04 | hypothetical protein | FIG00633360: hypothetical protein | - none - |
| EF2406 | *glyS* | 2.29 | 4,06E-04 | glycyl-tRNA synthetase subunit beta | Glycyl-tRNA synthetase beta chain (EC 6,1,1,14) | CBSS-349161,4,peg,2427; Glycyl-tRNA synthetase; glycyl-tRNA synthetase containing cluster; tRNA aminoacylation, Gly |
| EF2407 | *glyQ* | 2.5 | 3,02E-04 | glycyl-tRNA synthetase subunit alpha | Glycyl-tRNA synthetase alpha chain (EC 6,1,1,14) | CBSS-349161,4,peg,2427; glycyl-tRNA synthetase; glycyl-tRNA synthetase containing cluster; tRNA aminoacylation, Gly |
| EF2471 | *argS* | 3.71 | 1,60E-04 | arginyl-tRNA synthetase | Arginyl-tRNA synthetase (EC 6,1,1,19) | tRNA aminoacylation, Arg |
| EF2731 | *rpmG-2* | -2.97 | 9,93E-04 | 50S ribosomal protein L33 | LSU ribosomal protein L33p @ LSU ribosomal protein L33p, zinc-dependent | Ribosome LSU bacterial; ribosome LSU bacterial |
| EF3292 | *serS-2* | 2.15 | 8,88E-04 | seryl-tRNA synthetase | Seryl-tRNA synthetase (EC 6,1,1,11) | Glycine and serine utilization; tRNA aminoacylation, Ser |
| **[K] Transcription** | | | | | | |
| EF0102 | *-* | 2.12 | 9,33E-04 | ArgR family transcriptional regulator | Arginine pathway regulatory protein ArgR, repressor of arg regulon | Arginine deiminase pathway; arginine and ornithine degradation |
| EF0103 | *-* | 2.11 | 9,36E-04 | ArgR family transcriptional regulator | Arginine pathway regulatory protein ArgR, repressor of arg regulon | Arginine deiminase pathway; arginine and ornithine degradation |
| EF0112 | *-* | 2.85 | 1,87E-04 | hypothetical protein | FIG00630877: hypothetical protein | - none - |
| EF0113 | *-* | 3.72 | 6,85E-05 | hypothetical protein | hypothetical protein | - none - |
| EF0403 | *-* | -2.4 | 8,47E-04 | MarR family transcriptional regulator | Transcriptional regulator, MarR family | - none - |
| EF0571 | *-* | 2.7 | 7,32E-04 | DNA-binding response regulator | DNA-binding response regulator KdpE | - none - |
| EF0601 | *-* | -2.87 | 1,62E-04 | TetR family transcriptional regulator | hypothetical protein | - none - |
| EF0607 | *-* | 2.81 | 3,12E-04 | ParB-like nuclease domain-containing protein | Co-activator of prophage gene expression IbrB | IbrA and IbrB: co-activators of prophage gene expression |
| EF0719 | *-* | 38.87 | 9,30E-06 | DeoR family transcriptional regulator | Transcriptional repressor of the fructose operon, DeoR family | Fructose utilization |
| EF0731 | *aguR* | 2.48 | 6,81E-04 | LuxR family transcriptional regulator | Transcriptional regulator, luxR family, associated with agmatine catabolism | Polyamine metabolism |
| EF0781 | *-* | -7.55 | 4,82E-04 | cold shock domain-contain protein | Cold shock protein CspA | Cold shock, CspA family of proteins |
| EF0828 | *-* | 3.07 | 1,30E-04 | hypothetical protein | Transcriptional antiterminator of lichenan operon, BglG family | Beta-glucoside metabolism |
| EF0869 | *-* | -2.7 | 6,18E-04 | Cro/CI family transcriptional regulator | DNA-binding protein | - none - |
| EF1156 | *-* | 3.68 | 2,08E-04 | GntR family transcriptional regulator | Transcriptional regulator | - none - |
| EF1210 | *-* | 2.53 | 2,53E-04 | response regulator | Two-component response regulator, malate (EC 2,7,3,-) | Pyruvate metabolism I: anaplerotic reactions, PEP |
| EF1302 | *-* | 2.09 | 5,98E-04 | transcriptional regulator | COG0583: Transcriptional regulator | - none - |
| EF1306 | *hrcA* | 4.8 | 1,01E-04 | heat-inducible transcription repressor | Heat-inducible transcription repressor HrcA | Heat shock dnaK gene cluster extended |
| EF1357 | *-* | 2 | 7,77E-04 | AraC family transcriptional regulator | two-component response regulator | - none - |
| EF1367 | *-* | -5.88 | 3,94E-04 | cold-shock domain-contain protein | Cold shock protein CspA | Cold shock, CspA family of proteins |
| EF1579 | *lexA* | -2.69 | 3,11E-04 | LexA repressor | SOS-response repressor and protease LexA (EC 3,4,21,88) | DNA repair, bacterial |
| EF1645 | *codY* | 2.71 | 1,82E-04 | transcriptional repressor CodY | GTP-sensing transcriptional pleiotropic repressor codY | Conserved gene cluster associated with Met-tRNA formyltransferase |
| EF1656 | *-* | 2.46 | 3,28E-04 | LysR family transcriptional regulator | Transcriptional regulator, LysR family | - none - |
| EF1809 | *-* | 2.31 | 4,09E-04 | GntR family transcriptional regulator | Predicted transcriptional regulator of N-Acetylglucosamine utilization, GntR family | Chitin and N-acetylglucosamine utilization |
| EF1965 | *-* | -4.29 | 4,63E-05 | SorC family transcriptional regulator | Central glycolytic genes regulator | - none - |
| EF1991 | *cspC* | -10.86 | 3,36E-04 | cold shock protein CspC | Cold shock protein CspA | Cold shock, CspA family of proteins |
| EF2528 | *-* | 2.73 | 2,31E-04 | Cro/CI family transcriptional regulator | transcriptional regulator, Cro/CI family | - none - |
| EF2688 | *-* | -2.49 | 4,04E-04 | Snf2 family protein | COG0553: Superfamily II DNA/RNA helicases, SNF2 family | - none - |
| EF2886 | *-* | -3.09 | 3,86E-04 | MarR family transcriptional regulator | Transcriptional regulator of fatty acid biosynthesis FabT | Fatty acid biosynthesis FASII |
| EF2958 | *-* | 2.19 | 4,15E-04 | LysR family transcriptional regulator | transcriptional regulator, LysR family | - none - |
| EF2966 | *-* | 14.02 | 3,67E-05 | BglG family transcriptional antiterminator | putative phosphotransferase enzyme II, A component( EC:2,7,1,69 ) | - none - |
| EF3126 | *rpoZ* | -2.96 | 6,45E-04 | DNA-directed RNA polymerase subunit omega | DNA-directed RNA polymerase omega subunit (EC 2,7,7,6) | CBSS-176299,4,peg,1292; RNA polymerase bacterial |
| EF3156 | *-* | -2.88 | 1,90E-04 | GntR family transcriptional regulator | Trehalose operon transcriptional repressor | Trehalose uptake and utilization |
| EF3175 | *-* | -2.78 | 4,05E-04 | rrf2 family protein | Rrf2 family transcriptional regulator, group III | Rrf2 family transcriptional regulators |
| EF3237 | *rpoC* | -2.02 | 6,20E-04 | DNA-directed RNA polymerase subunit beta' | DNA-directed RNA polymerase beta' subunit (EC 2,7,7,6) | Mycobacterium virulence operon involved in DNA transcription; RNA polymerase bacterial |
| **[L] Replication, recombination and repair** | | | | | | |
| EF0002 | *dnaN* | 2.57 | 3,31E-04 | DNA polymerase III subunit beta | DNA polymerase III beta subunit (EC 2,7,7,7) | DNA-replication; DNA replication cluster 1 |
| EF0257 | *mfd* | 2.46 | 8,42E-04 | transcription-repair coupling factor | Transcription-repair coupling factor | Cell division-ribosomal stress proteins cluster; DNA-replication; transcription factors bacterial; transcription repair cluster |
| EF0258 | *-* | 2.42 | 5,47E-04 | polysaccharide biosynthesis family protein | FIG006789: Stage V sporulation protein; Low temperature requirement B protein | Cell division-ribosomal stress proteins cluster |
| EF1406 | *uvrC* | -2.77 | 3,08E-04 | excinuclease ABC subunit C | Excinuclease ABC subunit C | DNA repair, UvrABC system |
| EF1545 | *recQ-1* | -3.01 | 2,38E-04 | ATP-dependent DNA helicase RecQ | ATP-dependent DNA helicase RecQ | DNA-replication; DNA repair, bacterial RecFOR pathway |
| EF2145 | *-* | 2.27 | 3,82E-04 | phage integrase family site specific recombinase | site-specific recombinase, phage integrase family | - none - |
| EF2855 | *-* | 2.32 | 3,77E-04 | phage integrase family site specific recombinase | site-specific recombinase, phage integrase family | - none - |
| **[M] Cell wall/membrane/envelope biogenesis** | | | | | | |
| EF0417 | *-* | 3.07 | 1,77E-04 | hypothetical protein | FIG00628419: hypothetical protein | - none - |
| EF0989 | *mraW* | -2.14 | 9,76E-04 | S-adenosyl-methyltransferase MraW | rRNA small subunit methyltransferase H | 16S rRNA modification within P site of ribosome; bacterial cell division |
| EF1172 | *-* | -3.35 | 2,15E-04 | teichoic acid biosynthesis protein B | CDP-glycerol: N-acetyl-beta-D-mannosaminyl-1,4-N-acetyl-D-glucosaminyldiphosphoundecaprenyl glycerophosphotransferase | Teichoic and lipoteichoic acids biosynthesis |
| EF1672 | *-* | 2.01 | 7,07E-04 | permease | FIG00628701: hypothetical protein | - none - |
| EF1810 | *gspA-1* | 3.98 | 8,06E-05 | general stress protein A | general stress protein A | - none - |
| EF1811 | *gspA-2* | 2.23 | 3,43E-04 | general stress protein A | general stress protein A | - none - |
| EF2151 | *glmS* | 2.21 | 4,67E-04 | glucosamine--fructose-6-phosphate aminotransferase | Glucosamine--fructose-6-phosphate aminotransferase [isomerizing] (EC 2,6,1,16) | Sialic acid metabolism; UDP-N-acetylmuramate from fructose-6-phosphate biosynthesis |
| EF2489 | *-* | 2.28 | 4,45E-04 | MurB family protein | MurB family protein | - none - |
| EF2524 | *-* | 3.2 | 1,47E-04 | sortase | Sortase A, LPXTG specific | Heme, hemin uptake and utilization systems in Gram positives; sortase |
| EF2585 | *-* | -2.21 | 8,58E-04 | mur ligase | proposed amino acid ligase found clustered with an amidotransferase | - none - |
| EF2605 | *murAA* | -2.2 | 8,85E-04 | UDP-N-acetylglucosamine 1-carboxyvinyltransferase 1 | UDP-N-acetylglucosamine 1-carboxyvinyltransferase (EC 2,5,1,7) | Peptidoglycan biosynthesis; UDP-N-acetylmuramate from fructose-6-phosphate biosynthesis |
| EF2917 | *-* | 2.28 | 4,60E-04 | UDP-N-acetylglucosamine 2-epimerase | UDP-N-acetylglucosamine 2-epimerase (EC 5,1,3,14) | Sialic Acid Metabolism |
| **[O] Posttranslational modification, protein turnover, chaperones** | | | | | | |
| EF0401 | *pcp* | 2.42 | 4,27E-04 | pyrrolidone-carboxylate peptidase | Pyrrolidone-carboxylate peptidase (EC 3,4,19,3) | Omega peptidases (EC 3,4,19,-) |
| EF0473 | *nrdH* | -2.81 | 3,67E-04 | ribonucleoside-diphosphate reductase 2, NrdH-redoxin | Glutaredoxin-like protein NrdH, required for reduction of Ribonucleotide reductase class Ib | Glutaredoxins; glutathione: redox cycle; ribonucleotide reduction |
| EF0770 | *-* | 2.76 | 7,04E-04 | hypothetical protein | FIG00628706: hypothetical protein | - none - |
| EF1307 | *grpE* | 7.45 | 1,06E-04 | heat shock protein GrpE | Heat shock protein GrpE | Heat shock dnaK gene cluster extended; protein chaperones |
| EF1308 | *dnaK* | 5.82 | 4,70E-05 | dnak protein | Chaperone protein DnaK | Heat shock dnaK gene cluster extended; protein chaperones |
| EF1310 | *dnaJ* | 2.48 | 4,07E-04 | dnaJ protein | Chaperone protein DnaJ | Heat shock dnaK gene cluster extended; protein chaperones |
| EF1338 | *trxB* | -2.17 | 8,52E-04 | thioredoxin reductase | Thioredoxin reductase (EC 1,8,1,9) | Thioredoxin-disulphide reductase; pyrimidine conversions |
| EF1646 | *hslU* | 2.96 | 3,47E-04 | ATP-dependent protease ATP-binding protein HslU | ATP-dependent hsl protease ATP-binding subunit HslU | Proteasome bacterial; proteolysis in bacteria, ATP-dependent |
| EF2355 | *clpB* | 2.55 | 5,17E-04 | ATP-dependent Clp protease, ATP-binding protein ClpB | ClpB protein | Protein chaperones; proteolysis in bacteria, ATP-dependent |
| EF2633 | *groEL* | 4.16 | 8,69E-05 | chaperonin, 60 kDa | Heat shock protein 60 family chaperone GroEL | GroEL GroES |
| EF2634 | *groES* | 3.31 | 1,88E-04 | chaperonin, 10 kDa | Heat shock protein 60 family co-chaperone GroES | GroEL GroES |
| EF2932 | *-* | 2.17 | 6,29E-04 | AhpC/TSA family protein | Thiol peroxidase, Tpx-type (EC 1,11,1,15) | CBSS-257314,1,peg,752; Thioredoxin-disulphide reductase |
| EF3164 | *-* | -2.54 | 3,83E-04 | methionine sulphoxide reductase B | Peptide methionine sulphoxide reductase MsrB (EC 1,8,4,12) | Peptide methionine sulphoxide reductase |
| **[P] Inorganic ion transport and metabolism** | | | | | | |
| EF0191 | *-* | -2.22 | 5,52E-04 | ferrichrome ABC transporter ATP-binding protein | Ferrichrome transport ATP-binding protein FhuC (TC 3,A,1,14,3) | - none - |
| EF0567 | *kdpA* | 5.74 | 7,68E-05 | potassium-transporting ATPase subunit A | Potassium-transporting ATPase A chain (EC 3,6,3,12) (TC 3,A,3,7,1) | Potassium homeostasis |
| EF0568 | *kdpB* | 5.02 | 8,82E-05 | potassium-transporting ATPase subunit B | Potassium-transporting ATPase B chain (EC 3,6,3,12) (TC 3,A,3,7,1) | Potassium homeostasis |
| EF0569 | *kdpC* | 3.18 | 1,67E-04 | potassium-transporting ATPase subunit C | Potassium-transporting ATPase C chain (EC 3,6,3,12) (TC 3,A,3,7,1) | Potassium homeostasis |
| EF0758 | *-* | -2.82 | 2,68E-04 | cadmium-translocating P-type ATPase | Lead, cadmium, zinc and mercury transporting ATPase (EC 3,6,3,3) (EC 3,6,3,5); Copper-translocating P-type ATPase (EC 3,6,3,4) | Copper transport system; copper homeostasis |
| EF0872 | *-* | 2.01 | 7,26E-04 | potassium uptake protein | Kup system potassium uptake protein | Potassium homeostasis |
| EF1054 | *-* | 2.47 | 2,57E-04 | ABC transporter permease | ABC transporter, permease protein | - none - |
| EF1525 | *-* | -2.71 | 9,16E-04 | FUR family transcriptional regulator | Ferric uptake regulation protein FUR | Bacterial RNA-metabolizing Zn-dependent hydrolases; oxidative stress |
| EF1641 | *-* | 2.32 | 5,63E-04 | iron ABC transporter iron compound-binding protein | Vitamin B12 ABC transporter, B12-binding component BtuF | - none - |
| EF2417 | *-* | -2.01 | 8,44E-04 | FUR family transcriptional regulator | Zinc uptake regulation protein ZUR | Glycyl-tRNA synthetase containing cluster; oxidative stress |
| EF2441 | *-* | 7.39 | 1,67E-05 | hypothetical protein | Phosphate transport regulator (distant homolog of PhoU) | Phosphate metabolism |
| EF2442 | *-* | 2.47 | 3,29E-04 | phosphate transporter family protein | Probable low-affinity inorganic phosphate transporter | Phosphate metabolism |
| EF2496 | *-* | 2.01 | 6,76E-04 | pheromone cOB1 /lipoprotein YaeC family | Methionine ABC transporter substrate-binding protein | Methionine biosynthesis; methionine degradation |
| EF3069 | *-* | -2.26 | 8,63E-04 | formate/nitrite transporter family protein | formate/nitrite transporter family protein | - none - |
| **[R] General function prediction only** | | | | | | |
| EF0048 | *-* | -2.49 | 5,56E-04 | hypothetical protein | Hypothetical protein DUF901, similar to C-terminal domain of ribosome protection-type Tc-resistance proteins | Conserved gene cluster possibly involved in RNA metabolism |
| EF0469 | *-* | 4.51 | 2,80E-04 | hypothetical protein | Beta-propeller domains of methanol dehydrogenase type | - none - |
| EF0491 | *-* | -2.11 | 9,41E-04 | hypothetical protein | conserved domain protein | - none - |
| EF0559 | *-* | -2.16 | 6,18E-04 | polysaccharide biosynthesis family protein | Polysaccharide biosynthesis protein | - none - |
| EF0827 | *-* | 2.52 | 2,86E-04 | Gfo/Idh/MocA family oxidoreductase | aerobic energy metabolism | - none - |
| EF1041 | *-* | 9.01 | 5,88E-05 | xanthine/uracil permeases family protein | Xanthine/uracil/thiamine/ascorbate permease family protein | Purine utilization |
| EF1244 | *-* | 5.19 | 1,05E-04 | Gfo/Idh/MocA family oxidoreductase | Oxidoreductase (EC 1,1,1,-) | - none - |
| EF1510 | *-* | -2.38 | 6,22E-04 | hypothetical protein | probably aromatic ring hydroxylating enzyme, evidenced by COGnitor; PaaD-like protein (DUF59) involved in Fe-S cluster assembly | Iron-sulphur cluster assembly |
| EF1549 | *engA* | -2.57 | 3,08E-04 | GTP-binding protein EngA | GTP-binding protein EngA | Ribosome post-transcriptional modification and chromosomal segregation cluster; universal GTPases |
| EF1826 | *adhA* | 2.87 | 1,90E-04 | alcohol dehydrogenase | Alcohol dehydrogenase (EC 1,1,1,1) | 5-FCL-like protein; fermentations: mixed acid; glycerolipid and Glycerophospholipid metabolism in bacteria |
| EF1878 | *-* | -2.02 | 8,43E-04 | ATP/GTP-binding protein | FIG00627334: hypothetical protein | - none - |
| EF2569 | *-* | 2.07 | 6,63E-04 | hypothetical protein | CTP:molybdopterin cytidylyltransferase | Xanthine dehydrogenase subunits |
| EF2586 | *-* | -2.78 | 4,50E-04 | cobyric acid synthase | Putative amidotransferase similar to cobyric acid synthase | - none - |
| EF2662 | *-* | 2.31 | 7,70E-04 | choline binding protein | putative choline binding protein | - none - |
| EF2692 | *-* | -2.57 | 3,46E-04 | hypothetical protein | tRNA (adenine37-N(6))-methyltransferase TrmN6 (EC 2,1,1,223) | RNA methylation |
| EF2720 | *-* | -9.18 | 1,17E-05 | ABC transporter ATP-binding protein | ABC transporter, ATP-binding protein | - none - |
| EF2732 | *-* | 2.17 | 9,69E-04 | hypothetical protein | FIG00630165: hypothetical protein | - none - |
| EF3312 | *trmE* | -3.04 | 2,62E-04 | tRNA modification GTPase TrmE | GTPase and tRNA-U34 5-formylation enzyme TrmE | RNA modification and chromosome partitioning cluster; RNA modification cluster; universal GTPases; mnm5U34 biosynthesis bacteria; tRNA modification Bacteria |
| **[S] Function unknown** | | | | | | |
| EF0050 | *-* | -6.01 | 4,57E-05 | hypothetical protein | Veg protein | - none - |
| EF0054 | *-* | 3.01 | 8,10E-04 | hypothetical protein | hypothetical protein | - none - |
| EF0083 | *-* | -4.16 | 9,63E-04 | hypothetical protein | FIG00627510: hypothetical protein | - none - |
| EF0095 | *-* | 4.16 | 1,08E-04 | lipoprotein | lipoprotein, putative | - none - |
| EF0129 | *-* | -2.02 | 9,27E-04 | Cro/CI family transcriptional regulator | Ans operon transcriptional repressor | - none - |
| EF0131 | *-* | 4.64 | 4,09E-05 | hypothetical protein | FIG00632803: hypothetical protein | - none - |
| EF0132 | *-* | 5.78 | 4,31E-05 | hypothetical protein | FIG00632933: hypothetical protein | - none - |
| EF0133 | *-* | 3.14 | 1,87E-04 | hypothetical protein | FIG00628307: hypothetical protein | - none - |
| EF0134 | *-* | 2.04 | 9,98E-04 | hypothetical protein | FIG00628114: hypothetical protein | - none - |
| EF0235 | *-* | 2.13 | 7,26E-04 | hypothetical protein | membrane protein | - none - |
| EF0241 | *-* | 2.09 | 7,57E-04 | hypothetical protein | PlcB, ORFX, ORFP, ORFB, ORFA, ldh gene | - none - |
| EF0261 | *-* | -2.51 | 3,12E-04 | hypothetical protein | hypothetical protein | - none - |
| EF0288 | *-* | 7.44 | 3,28E-05 | hypothetical protein | FIG00629202: hypothetical protein | - none - |
| EF0304 | *-* | 2.72 | 3,45E-04 | lipoprotein | lipoprotein, putative | - none - |
| EF0311 | *-* | 2.37 | 3,49E-04 | hypothetical protein | FIG00633107: hypothetical protein | - none - |
| EF0312 | *-* | 2.34 | 3,06E-04 | aspartate 1-decarboxylase domain-containing protein | aspartate 1-decarboxylase domain protein | - none - |
| EF0316 | *-* | 2.07 | 5,92E-04 | hypothetical protein | hypothetical protein | - none - |
| EF0317 | *-* | 2.32 | 4,52E-04 | Cro/CI family transcriptional regulator | transcriptional regulator, Cro/CI family | - none - |
| EF0321 | *-* | 2.54 | 1,98E-04 | hypothetical protein | FIG00631564: hypothetical protein | - none - |
| EF0323 | *-* | 2.8 | 1,59E-04 | hypothetical protein | FIG00630221: hypothetical protein | - none - |
| EF0333 | *-* | 2.82 | 1,71E-04 | hypothetical protein | conserved hypothetical protein TIGR01630 | - none - |
| EF0337 | *-* | 2.73 | 2,78E-04 | hypothetical protein | hypothetical protein | - none - |
| EF0338 | *-* | 3.81 | 2,71E-04 | scaffold protein | Phage capsid and scaffold | Phage capsid proteins |
| EF0339 | *-* | 2.98 | 1,24E-04 | major capsid protein | Phage major capsid protein | Phage capsid proteins |
| EF0340 | *-* | 3.08 | 1,20E-04 | hypothetical protein | hypothetical protein | - none - |
| EF0341 | *-* | 2.92 | 1,80E-04 | hypothetical protein | FIG00633406: hypothetical protein | - none - |
| EF0342 | *-* | 3.12 | 1,37E-04 | hypothetical protein | phage head-tail adaptor, putative | - none - |
| EF0343 | *-* | 2.99 | 2,94E-04 | hypothetical protein | conserved hypothetical protein TIGR01725 | - none - |
| EF0344 | *-* | 2.11 | 7,70E-04 | hypothetical protein | hypothetical protein | - none - |
| EF0345 | *-* | 4.03 | 6,72E-05 | hypothetical protein | conserved domain protein | - none - |
| EF0347 | *-* | 3.18 | 1,48E-04 | peptide methionine sulphoxide reductase domain-containing protein | peptide methionine sulphoxide reductase domain protein | - none - |
| EF0350 | *-* | 2.05 | 8,28E-04 | hypothetical protein | FIG00628636: hypothetical protein | - none - |
| EF0351 | *-* | 2.44 | 3,42E-04 | structural protein | Phage baseplate protein | Phage baseplate proteins |
| EF0354 | *-* | 2.29 | 3,50E-04 | holin | holin | - none - |
| EF0439 | *-* | 3.17 | 2,80E-04 | immunity protein PlnM | FIG00628214: hypothetical protein | - none - |
| EF0468 | *-* | 5.54 | 2,62E-04 | LemA family protein | LemA family protein | - none - |
| EF0485 | *-* | -2.57 | 6,15E-04 | aggregation substance | Aggregation substance Asa1/PrgB | Sex pheromones in *Enterococcus faecalis* and other Firmicutes |
| EF0487 | *-* | -2.4 | 3,26E-04 | hypothetical protein | FIG00630890: hypothetical protein | - none - |
| EF0488 | *-* | -2.36 | 8,47E-04 | hypothetical protein | orf6 | - none - |
| EF0492 | *-* | -2.29 | 9,28E-04 | hypothetical protein | FIG00631362: hypothetical protein | - none - |
| EF0523 | *-* | 2.44 | 3,32E-04 | hypothetical protein | FIG00632739: hypothetical protein | - none - |
| EF0525 | *-* | 3.9 | 5,29E-05 | cylL-L protein | hypothetical protein | - none - |
| EF0526 | *-* | 5.15 | 2,14E-04 | cylL-S protein | hypothetical protein | - none - |
| EF0604 | *-* | 2.19 | 9,78E-04 | gls24 protein | General stress protein, Gls24 family | - none - |
| EF0608 | *-* | 2.74 | 2,72E-04 | hypothetical protein |  |  |
| EF0778 | *-* | -2.12 | 8,96E-04 | hypothetical protein | FIG00628137: hypothetical protein | - none - |
| EF0797 | *-* | 2.48 | 6,72E-04 | hypothetical protein | FIG00629788: hypothetical protein | - none - |
| EF0905 | *-* | 2.26 | 4,70E-04 | pentapeptide repeat-containing protein | pentapeptide repeat family protein | - none - |
| EF0906 | *-* | 2.26 | 3,62E-04 | hypothetical protein | FIG00628713: hypothetical protein | - none - |
| EF0971 | *-* | -2.94 | 1,29E-04 | phosphate-starvation-inducible protein PsiE | phosphate-starvation-inducible protein PsiE | - none - |
| EF0987 | *-* | -2.47 | 5,95E-04 | lipoprotein |  |  |
| EF0988 | *-* | -2.73 | 9,78E-04 | cell division protein MraZ | Cell division protein MraZ | 16S rRNA modification within P site of ribosome; bacterial cell division; bacterial cytoskeleton |
| EF1001 | *-* | -2.46 | 7,92E-04 | S4 domain-containing protein | FIG001583: hypothetical protein, contains S4-like RNA binding domain | Cell division cluster |
| EF1035 | *-* | 2.75 | 1,46E-04 | lipoprotein | lipoprotein, putative | - none - |
| EF1107 | *-* | 3.02 | 1,20E-04 | hypothetical protein |  |  |
| EF1168 | *-* | -9.66 | 1,92E-04 | hypothetical protein |  |  |
| EF1202 | *-* | -2.09 | 6,09E-04 | hypothetical protein | Hypothetical protein possible functionally linked with Alanyl-tRNA synthetase | CBSS-279010,5,peg,3195 |
| EF1250 | *-* | -2.06 | 8,06E-04 | hypothetical protein | conserved hypothetical protein | - none - |
| EF1282 | *-* | 2.19 | 3,75E-04 | hypothetical protein | FIG00629006: hypothetical protein | - none - |
| EF1283 | *-* | 2.37 | 5,65E-04 | RinA family transcriptional regulator | Integrase regulator RinA | - none - |
| EF1284 | *-* | 2.61 | 2,46E-04 | structural protein | FIG00628002: hypothetical protein | - none - |
| EF1285 | *-* | 3.09 | 2,25E-04 | major tail protein | Phage tail protein | Phage tail proteins |
| EF1286 | *-* | 2.28 | 4,70E-04 | hypothetical protein | FIG00630610: hypothetical protein | - none - |
| EF1309 | *-* | 4.72 | 8,67E-05 | hypothetical protein | FIG00629320: hypothetical protein | - none - |
| EF1324 | *-* | -4.44 | 1,94E-04 | hypothetical protein | FIG00628040: hypothetical protein | - none - |
| EF1346 | *-* | 2.71 | 2,34E-04 | hypothetical protein | FIG00632988: hypothetical protein | - none - |
| EF1359 | *-* | 11.31 | 3,17E-05 | hypothetical protein | Phosphoenolpyruvate-dihydroxyacetone phosphotransferase (EC 2,7,1,121), subunit DhaM; DHA-specific IIA component | Dihydroxyacetone kinases; Dihydroxyacetone kinases |
| EF1368 | *-* | 2.91 | 3,99E-04 | hypothetical protein | FIG00627827: hypothetical protein | - none - |
| EF1402 | *-* | -2.53 | 3,00E-04 | hypothetical protein | FIG00629133: hypothetical protein | - none - |
| EF1487 | *-* | -2.19 | 8,33E-04 | hypothetical protein | FIG00632154: hypothetical protein | - none - |
| EF1734 | *-* | -3.04 | 4,54E-04 | hypothetical protein | FIG00627214: hypothetical protein | - none - |
| EF1753 | *-* | 2.28 | 9,30E-04 | hypothetical protein | Hypothetical protein, homolog of fig\|393130,3,peg,2627 | - none - |
| EF1796 | *-* | 3.21 | 1,39E-04 | lipoprotein | lipoprotein, putative | - none - |
| EF1820 | *-* | -4.24 | 5,02E-04 | histidine kinase | Histidine kinase of the competence regulon ComD | - none - |
| EF1825 | *-* | 2.72 | 2,04E-04 | hypothetical protein | conserved domain protein | - none - |
| EF1909 | *-* | -2.36 | 5,86E-04 | hypothetical protein | hypothetical protein | - none - |
| EF1946 | *-* | -3.31 | 1,32E-04 | hypothetical protein |  |  |
| EF1947 | *-* | -3.12 | 1,07E-04 | hypothetical protein | FIG00627452: hypothetical protein | - none - |
| EF1959 | *-* | -2.17 | 4,41E-04 | hypothetical protein | FIG00629473: hypothetical protein | - none - |
| EF2205 | *-* | 2.26 | 5,33E-04 | hypothetical protein | DUF1696 domain-containing protein | - none - |
| EF2405 | *-* | 2.26 | 6,21E-04 | hypothetical protein | hypothetical protein | - none - |
| EF2488 | *-* | 2.02 | 8,27E-04 | lipoprotein | lipoprotein, putative | - none - |
| EF2514 | *-* | 2.37 | 3,20E-04 | hypothetical protein | FIG00631877: hypothetical protein | - none - |
| EF2519 | *-* | 2.43 | 6,46E-04 | hypothetical protein | FIG00630351: hypothetical protein | - none - |
| EF2520 | *-* | 3.15 | 1,27E-04 | hypothetical protein | FIG00631614: hypothetical protein | - none - |
| EF2521 | *-* | 2.95 | 2,03E-04 | hypothetical protein | FIG00628325: hypothetical protein | - none - |
| EF2522 | *-* | 2.52 | 3,22E-04 | hypothetical protein | FIG00630862: hypothetical protein | - none - |
| EF2523 | *-* | 2.24 | 7,33E-04 | hypothetical protein | FIG00627501: hypothetical protein | - none - |
| EF2525 | *-* | 3.21 | 1,42E-04 | cell wall surface anchor family protein | FIG00632822: hypothetical protein | - none - |
| EF2526 | *-* | 2.88 | 3,81E-04 | hypothetical protein | FIG00631350: hypothetical protein | - none - |
| EF2529 | *-* | 3.86 | 7,39E-05 | hypothetical protein | FIG00629110: hypothetical protein | - none - |
| EF2530 | *-* | 4.11 | 7,22E-05 | hypothetical protein | hypothetical protein | - none - |
| EF2531 | *-* | 4.77 | 3,55E-05 | hypothetical protein | hypothetical protein | - none - |
| EF2532 | *-* | 3.96 | 1,10E-04 | hypothetical protein | FIG00629358: hypothetical protein | - none - |
| EF2534 | *-* | 4.77 | 6,69E-05 | hypothetical protein | FIG00629866: hypothetical protein | - none - |
| EF2536 | *-* | 3.45 | 1,04E-04 | hypothetical protein | hypothetical protein | - none - |
| EF2537 | *-* | 3.01 | 1,32E-04 | hypothetical protein | hypothetical protein | - none - |
| EF2539 | *-* | 3.36 | 1,39E-04 | hypothetical protein | FIG00628114: hypothetical protein | - none - |
| EF2540 | *-* | 4.37 | 1,43E-04 | hypothetical protein | FIG00628307: hypothetical protein | - none - |
| EF2541 | *-* | 5.38 | 4,45E-05 | hypothetical protein | FIG00632933: hypothetical protein | - none - |
| EF2542 | *-* | 5.38 | 2,54E-05 | hypothetical protein | FIG00632803: hypothetical protein | - none - |
| EF2547 | *-* | -3.89 | 1,14E-04 | hypothetical protein | FIG00628084: hypothetical protein | - none - |
| EF2548 | *-* | -2.67 | 4,49E-04 | hypothetical protein | FIG00628837: hypothetical protein | - none - |
| EF2606 | *-* | -7.84 | 1,16E-04 | hypothetical protein | FIG00627879: hypothetical protein | - none - |
| EF2619 | *-* | -2.14 | 4,20E-04 | hypothetical protein |  |  |
| EF2627 | *-* | -4.01 | 1,87E-04 | teichoic acid glycosylation protein | Teichoic acid glycosylation protein | Teichoic and lipoteichoic acids biosynthesis |
| EF2672 | *-* | -2.3 | 3,79E-04 | hypothetical protein | Adenylate cyclase | CBSS-222523,1,peg,1311 |
| EF2687 | *-* | -2.86 | 1,81E-04 | hypothetical protein | FIG00630846: hypothetical protein | - none - |
| EF2712 | *-* | 2.07 | 9,20E-04 | hypothetical protein |  |  |
| EF2713 | *-* | 3.6 | 1,20E-04 | cell wall surface anchor family protein | cell wall surface anchor family protein | - none - |
| EF2778 | *-* | 2.54 | 6,27E-04 | hypothetical protein | Substrate-specific component ThiW of predicted thiazole ECF transporter | 5-FCL-like protein; ECF class transporters; thiamin biosynthesis |
| EF2798 | *-* | -2.19 | 4,41E-04 | hypothetical protein | FIG00629228: hypothetical protein | - none - |
| EF2864 | *-* | 2.13 | 6,53E-04 | hypothetical protein | FIG00629262: hypothetical protein | - none - |
| EF2940 | *-* | 4.75 | 9,56E-05 | hypothetical protein | hypothetical protein | - none - |
| EF2941 | *-* | 4.67 | 8,41E-05 | hypothetical protein | hypothetical protein | - none - |
| EF2942 | *-* | 2.62 | 3,08E-04 | hypothetical protein | hypothetical protein | - none - |
| EF2944 | *-* | 2.52 | 3,11E-04 | hypothetical protein | hypothetical protein | - none - |
| EF2947 | *-* | 2.12 | 5,12E-04 | hypothetical protein | conserved hypothetical protein | - none - |
| EF2950 | *-* | 2.03 | 9,97E-04 | hypothetical protein | hypothetical protein | - none - |
| EF2951 | *-* | 2.48 | 2,56E-04 | hypothetical protein | prophage ps3 protein 13 | - none - |
| EF2952 | *-* | 2.21 | 7,37E-04 | hypothetical protein | hypothetical protein | - none - |
| EF2953 | *-* | 2.06 | 9,34E-04 | hypothetical protein | hypothetical protein | - none - |
| EF2964 | *ulaA* | 2.69 | 5,49E-04 | PTS system ascorbate-specific transporter subunit IIC | Putative integral membrane protein | - none - |
| EF3052 | *-* | -2.53 | 4,15E-04 | hypothetical protein | conserved hypothetical protein | - none - |
| EF3055 | *-* | -2.8 | 2,25E-04 | hypothetical protein | FIG00627537: hypothetical protein | - none - |
| EF3130 | *-* | 2.02 | 6,35E-04 | hypothetical protein | conserved hypothetical protein | - none - |
| EF3176 | *-* | -2.59 | 5,93E-04 | hypothetical protein | FIG00632250: hypothetical protein | - none - |
| EF3185 | *-* | -2.49 | 4,26E-04 | hypothetical protein | FIG00628102: hypothetical protein | - none - |
| EF3186 | *-* | -2.57 | 5,04E-04 | hypothetical protein | extracellular protein | - none - |
| EF3187 | *-* | -2.6 | 2,61E-04 | cell wall surface anchor family protein | FIG00628160: hypothetical protein | - none - |
| EF3188 | *-* | -2.59 | 3,96E-04 | hypothetical protein | FIG00629184: hypothetical protein | - none - |
| EF3189 | *-* | -4.46 | 8,06E-05 | hypothetical protein |  |  |
| EF3271 | *-* | 2.23 | 6,50E-04 | hypothetical protein | FIG00627927: hypothetical protein | - none - |
| EF3287 | *-* | -4.02 | 1,07E-04 | hypothetical protein |  |  |
| EF3303 | *-* | 2.37 | 9,60E-04 | myosin-cross-reactive antigen | myosin-crossreactive antigen | - none - |
| EF3313 | *-* | -3.84 | 5,52E-05 | hypothetical protein |  |  |
| **[T] Signal transduction mechanisms** | | | | | | |
| EF0373 | *-* | 2.04 | 6,57E-04 | sensor histidine kinase | putative Two-component system sensor histidine kinase | - none - |
| EF0570 | *kdpD* | 3.17 | 2,38E-04 | sensor histidine kinase KdpD | Osmosensitive K+ channel histidine kinase KdpD (EC 2,7,3,-) | Potassium homeostasis |
| EF1084 | *-* | -2.01 | 9,13E-04 | universal stress protein | Universal stress protein family | CBSS-269801,1,peg,809 |
| EF1209 | *-* | 2.48 | 6,22E-04 | sensory box histidine kinase | Two-component sensor histidine kinase, malate (EC 2,7,3,-) | Pyruvate metabolism I: anaplerotic reactions, PEP |
| EF1982 | *-* | 3.89 | 1,68E-04 | universal stress protein | Universal stress protein family | CBSS-269801,1,peg,809 |
| **[U] Intracellular trafficking, secretion, and vesicular transport** | | | | | | |
| EF1583 | *-* | -3.15 | 3,06E-04 | N-acetylmuramoyl-L-alanine amidase | Membrane-bound lytic murein transglycosylase D precursor (EC 3,2,1,-) | Murein Hydrolases |
| EF2620 | *secG* | -3.29 | 2,78E-04 | preprotein translocase subunit SecG | Preprotein translocase subunit SecG (TC 3,A,5,1,1) | CBSS-331978,3,peg,2915; murein hydrolase regulation and cell death |
| EF2730 | *secE* | -5.3 | 2,65E-04 | preprotein translocase subunit SecE | Preprotein translocase subunit SecE (TC 3,A,5,1,1) | LSU ribosomal proteins cluster |
| **[V] Defense mechanisms** | | | | | | |
| EF0460 | *-* | 2.55 | 2,71E-04 | hypothetical protein | Beta-lactamase class C and other penicillin binding proteins | Beta-lactamase |
| EF1732 | *-* | -2.96 | 1,57E-04 | ABC transporter ATP-binding protein/peptidase | ABC transporter, ATP-binding/permease protein | - none - |
| EF1733 | *-* | -2.19 | 9,37E-04 | ABC transporter ATP-binding protein/peptidase | ABC transporter, ATP-binding/permease protein | - none - |
