# Supplementary material for: The Relationship among Tyrosine Decarboxylase and Agmatine Deiminase Pathways in Enterococcus faecalis
Source: Front Microbiol. 2017 Nov 1;8:2107. doi: 10.3389/fmicb.2017.02107 (PMC5672081; doi:10.3389/fmicb.2017.02107)
Supplement: Supplementary file 2 [file Table_2.DOCX]

Table S2: Gene set enrichment analysis results. Only pathways with an adjusted p value of <0.01 are shown. The number of genes present in *Enterococcus faecalis* V583 is indicated, as are the numbers and locus names of overexpressed and underexpressed genes.

| **KEGG pathway** | **Nº genes** | **Nº genes over expressed** | **Nº genes under expressed** | **Locus overexpressed** | **Locus underexpressed** |
| --- | --- | --- | --- | --- | --- |
| Glycolysis / gluconeogenesis | 22 | 4 | 0 | EF0900, EF1068, EF1503, EF1526 |  |
| Galactose metabolism | 30 | 5 | 1 | EF0718, EF1068, EF1069, EF1807, EF2445 | EF1603 |
| Pentose phosphate pathway | 25 | 4 | 1 | EF0174, EF1503, EF2961, EF3142 | EF2073 |
| Amino sugar and nucleotide sugar metabolism | 53 | 12 | 1 | EF0020, EF0677, EF0717, EF1018, EF1069, EF1836, EF2151, EF2425, EF2489, EF2863, EF2917, EF2966 | EF2605 |
| Purine metabolism | 60 | 18 | 5 | EF0002, EF0014, EF0186, EF0735, EF1036, EF1222, EF1777, EF1778, EF1779, EF1780, EF1781, EF1782, EF1783, EF1784, EF1785, EF1787, EF2429, EF2431 | EF2073, EF2672, EF3126, EF3127, EF3237 |
| Pyrimidine metabolism | 45 | 6 | 2 | EF0002, EF0173, EF0175, EF0186, EF1036, EF1147 | EF3126, EF3237 |
| Alanine, aspartate and glutamate metabolism | 11 | 4 | 0 | EF0014, EF1781, EF1783, EF2151 |  |
| Phenylalanine, tyrosine and tryptophan biosynthesis | 11 | 0 | 8 |  | EF1561, EF1562, EF1563, EF1564, EF1565, EF1566, EF1567, EF1568 |
| Aminoacyl-tRNA biosynthesis | 40 | 7 | 1 | EF1779, EF1970, EF1971, EF2406, EF2407, EF2471, EF3292 | EF0633 |
