# Supplementary material for: The Relationship among Tyrosine Decarboxylase and Agmatine Deiminase Pathways in Enterococcus faecalis
Source: Front Microbiol. 2017 Nov 1;8:2107. doi: 10.3389/fmicb.2017.02107 (PMC5672081; doi:10.3389/fmicb.2017.02107)
Supplement: Supplementary file 3 [file Image_1.PDF]

Figure S1: KEGG maps of metabolic pathways resulting after the Gene Set Enrichment Analysis for Prokaryotes (GSEA-Pro) analysis (adjusted p-values < 0.01). The pathway name is shown en each map. Green rectangles indicate genes present in *E. faecalis* V583 genome; black border: genes which expression was not modified, red border: over-expressed genes, light blue border: under-expressed genes.

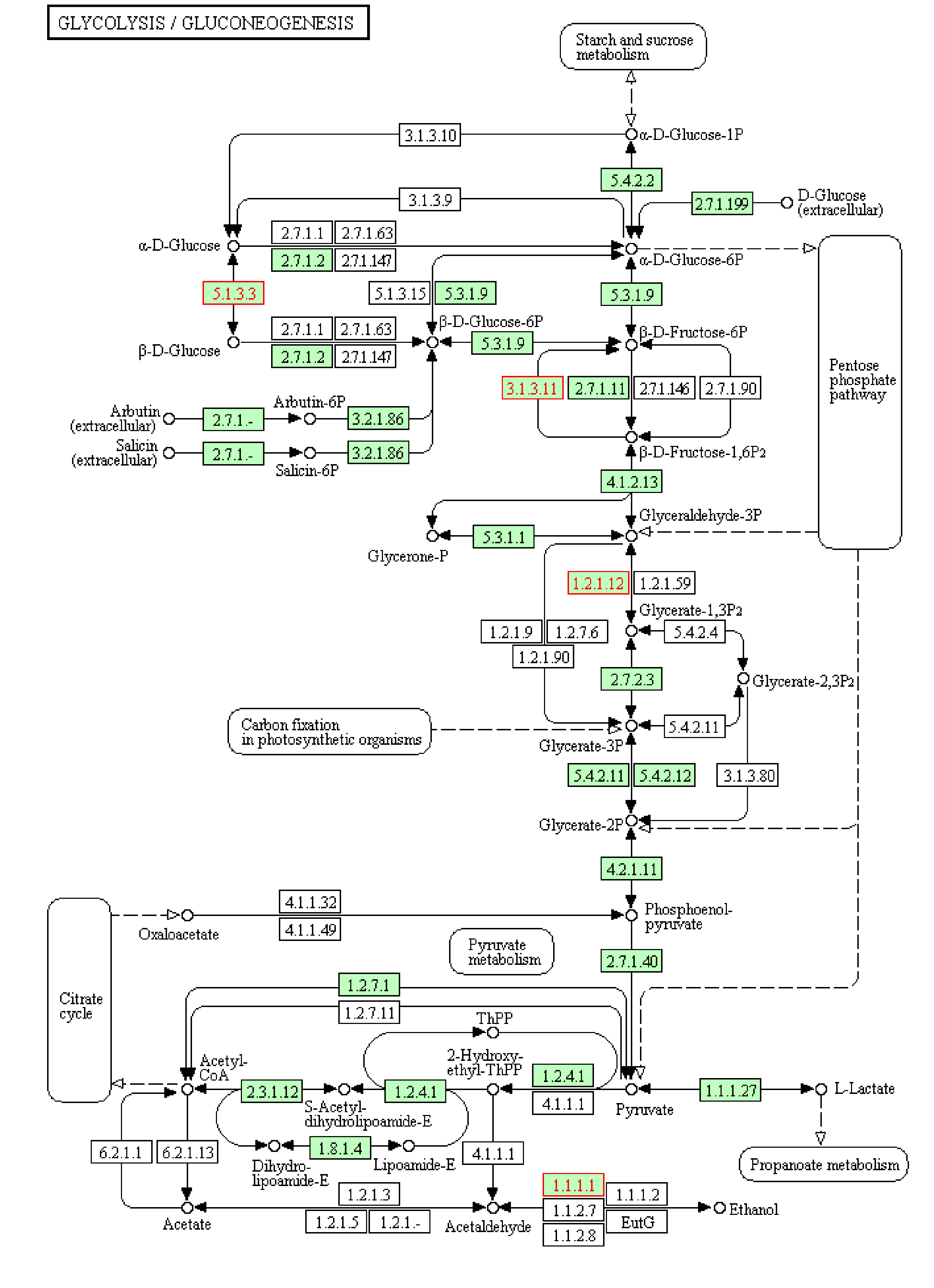
GLYCOLYSIS / GLUCONEOGENESIS
Starch and sucrose metabolism
α-D-Glucose-1P
3.1.3.10
5.4.2.2
3.1.3.9
2.7.1.199
D-Glucose (extracellular)
α-D-Glucose
2.7.1.1
2.7.1.63
2.7.1.2
2.7.1.147
α-D-Glucose-6P
5.1.3.3
5.1.3.15
5.3.1.9
β-D-Glucose
β-D-Glucose-6P
β-D-Fructose-6P
3.1.3.11
2.7.1.11
2.7.1.146
2.7.1.90
Pentose phosphate pathway
Arbutin (extracellular)
2.7.1.-
Arbutin-6P
3.2.1.86
Salicin (extracellular)
Salicin-6P
β-D-Fructose-1,6P2
4.1.2.13
Glyceraldehyde-3P
Glycerone-P
5.3.1.1
1.2.1.12
1.2.1.59
Glycerate-1,3P2
1.2.1.9
1.2.7.6
1.2.1.90
5.4.2.4
2.7.2.3
Glycerate-2,3P2
Carbon fixation in photosynthetic organisms
Glycerate-3P
5.4.2.11
5.4.2.12
3.1.3.80
Glycerate-2P
4.2.1.11
Citrate cycle
Oxaloacetate
4.1.1.32
4.1.1.49
Phosphoenol-pyruvate
Pyruvate metabolism
2.7.1.40
1.2.7.1
1.2.7.11
ThPP
Acetyl-CoA
2-Hydroxy-ethyl-ThPP
2.3.1.12
1.2.4.1
4.1.1.1
Pyruvate
1.1.1.27
L-Lactate
S-Acetyl-dihydrolipoamide-E
1.8.1.4
Dihydro-lipoamide-E
Lipoamide-E
6.2.1.1
6.2.1.13
Propanoate metabolism
1.2.1.3
1.1.1.1
1.1.1.2
Ethanol
Acetate
1.2.1.5
1.2.1.-
Acetaldehyde
1.1.2.7
EutG
1.1.2.8

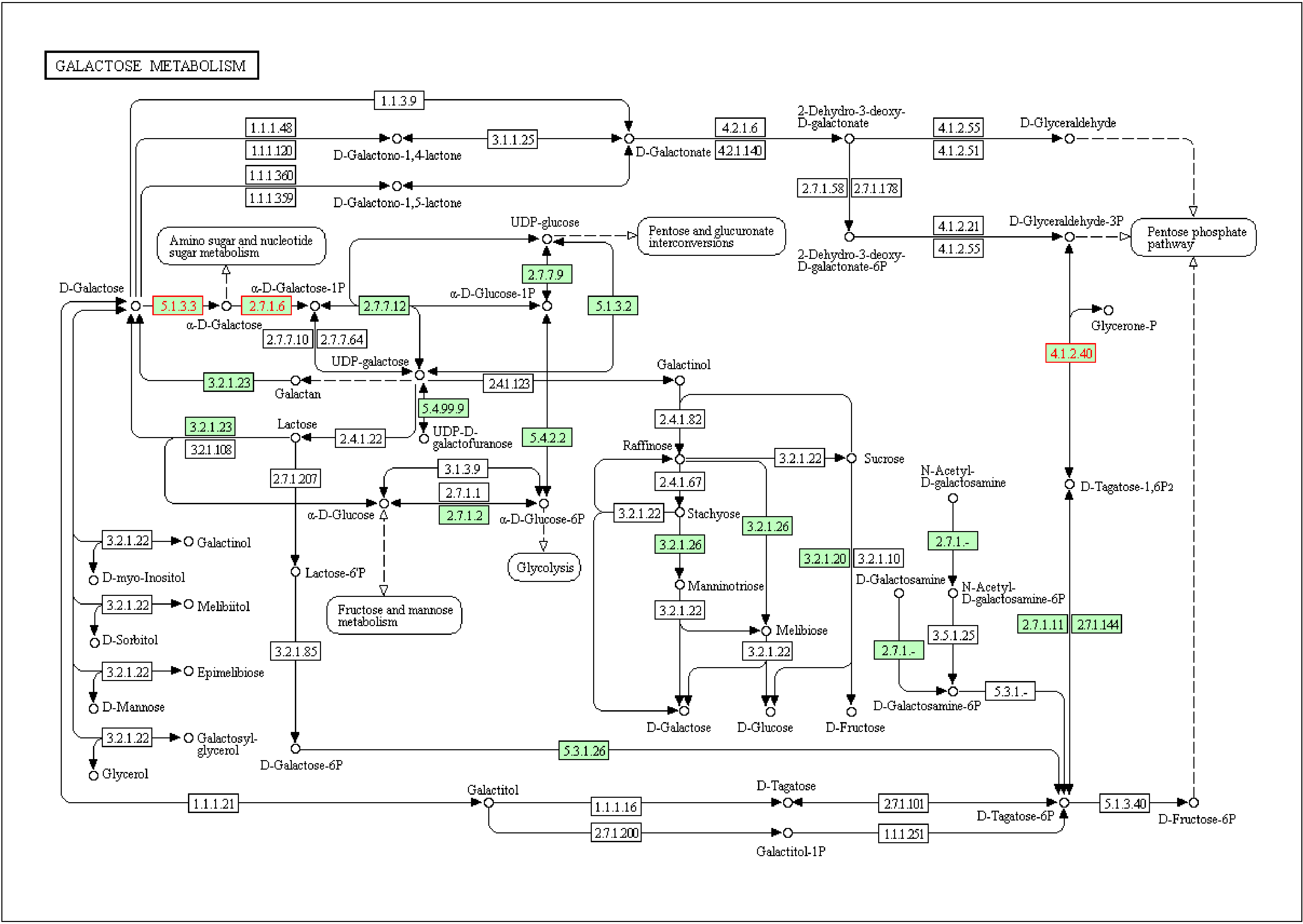
GALACTOSE METABOLISM
1.1.3.9
1.1.1.48
1.1.1.120
1.1.1.360
1.1.1.359
D-Galactono-1,4-lactone
3.1.1.25
D-Galactono-1,5-lactone
D-Galactonate
4.2.1.6
4.2.1.140
2-Dehydro-3-deoxy-D-galactonate
2.7.1.58
2.7.1.178
2-Dehydro-3-deoxy-D-galactonate-6P
4.1.2.55
4.1.2.51
D-Glyceraldehyde
4.1.2.21
4.1.2.55
D-Glyceraldehyde-3P
Pentose phosphate pathway
Amino sugar and nucleotide sugar metabolism
UDP-glucose
Pentose and glucuronate interconversions
D-Galactose
5.1.3.3
α-D-Galactose
2.7.1.6
α-D-Galactose-1P
2.7.7.12
2.7.7.10
2.7.7.64
2.7.7.9
α-D-Glucose-1P
5.1.3.2
UDP-galactose
3.2.1.23
Galactan
2.4.1.123
Galactinol
5.4.99.9
UDP-D-galactofuranose
5.4.2.2
3.2.1.23
3.2.1.108
Lactose
2.4.1.22
2.7.1.207
3.1.3.9
2.7.1.1
2.7.1.2
α-D-Glucose
α-D-Glucose-6P
Glycolysis
Fructose and mannose metabolism
Lactose-6P
3.2.1.85
D-Galactose-6P
5.3.1.26
2.4.1.82
Raffinose
3.2.1.22
Sucrose
2.4.1.67
3.2.1.22
Stachyose
3.2.1.26
3.2.1.26
Manninotriose
3.2.1.22
Melibiose
3.2.1.22
3.2.1.20
3.2.1.10
D-Galactose
D-Glucose
D-Fructose
N-Acetyl-D-galactosamine
2.7.1.-
D-Galactosamine
N-Acetyl-D-galactosamine-6P
3.5.1.25
2.7.1.-
D-Galactosamine-6P
5.3.1.-
2.7.1.11
2.7.1.144
4.1.2.40
Glycerone-P
D-Tagatose-1,6P2
3.2.1.22
Galactinol
D-myo-Inositol
3.2.1.22
Melibiitol
D-Sorbitol
3.2.1.22
Epimelibiose
D-Mannose
3.2.1.22
Galactosyl-glycerol
Glycerol
1.1.1.21
Galactitol
1.1.1.16
D-Tagatose
2.7.1.101
D-Tagatose-6P
5.1.3.40
D-Fructose-6P
2.7.1.200
Galactitol-1P
1.1.1.251

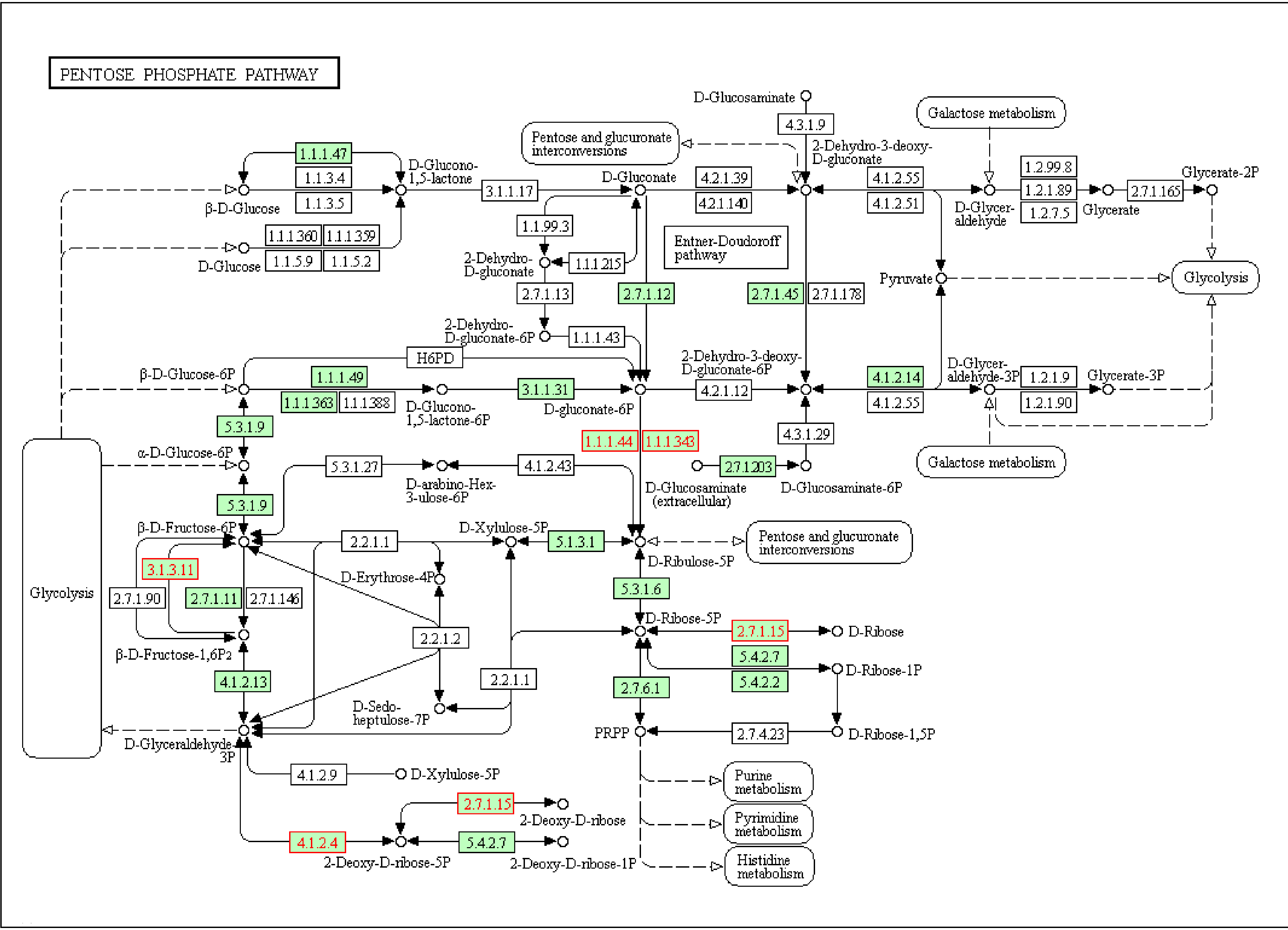
PENTOSE PHOSPHATE PATHWAY
D-Glucosaminate
4.3.1.9
Galactose metabolism
Pentose and glucuronate interconversions
2-Dehydro-3-deoxy-D-gluconate
1.1.1.47
1.1.3.4
1.1.3.5
D-Glucono-1,5-lactone
3.1.1.17
D-Gluconate
4.2.1.39
4.2.1.140
4.1.2.55
4.1.2.51
1.2.99.8
1.2.1.89
1.2.7.5
2.7.1.165
Glycerate-2P
β-D-Glucose
D-Glycer-aldehyde
Glycerate
1.1.1.360
1.1.1.359
1.1.99.3
Entner-Doudoroff pathway
1.1.5.9
1.1.5.2
D-Glucose
2-Dehydro-D-gluconate
1.1.1.215
Pyruvate
Glycolysis
2.7.1.13
2.7.1.12
2.7.1.45
2.7.1.178
2-Dehydro-D-gluconate-6P
1.1.1.43
H6PD
2-Dehydro-3-deoxy-D-gluconate-6P
D-Glycer-aldehyde-3P
β-D-Glucose-6P
1.1.1.49
3.1.1.31
4.2.1.12
4.1.2.14
1.2.1.9
Glycerate-3P
1.1.1.363
1.1.1.388
D-Glucono-1,5-lactone-6P
D-gluconate-6P
4.1.2.55
1.2.1.90
5.3.1.9
1.1.1.44
1.1.1.343
4.3.1.29
Galactose metabolism
α-D-Glucose-6P
5.3.1.27
4.1.2.43
2.7.1.203
D-arabino-Hex-3-ulose-6P
D-Glucosaminate (extracellular)
D-Glucosaminate-6P
5.3.1.9
β-D-Fructose-6P
2.2.1.1
D-Xylulose-5P
5.1.3.1
Pentose and glucuronate interconversions
D-Ribulose-5P
3.1.3.11
D-Erythrose-4P
Glycolysis
2.7.1.90
2.7.1.11
2.7.1.146
5.3.1.6
D-Ribose-5P
2.7.1.15
D-Ribose
2.2.1.2
β-D-Fructose-1,6P2
5.4.2.7
D-Ribose-1P
4.1.2.13
2.2.1.1
5.4.2.2
2.7.6.1
D-Sedo-heptulose-7P
PRPP
2.7.4.23
D-Ribose-1,5P
D-Glyceraldehyde-3P
4.1.2.9
D-Xylulose-5P
Purine metabolism
2.7.1.15
2-Deoxy-D-ribose
Pyrimidine metabolism
4.1.2.4
5.4.2.7
2-Deoxy-D-ribose-5P
2-Deoxy-D-ribose-1P
Histidine metabolism

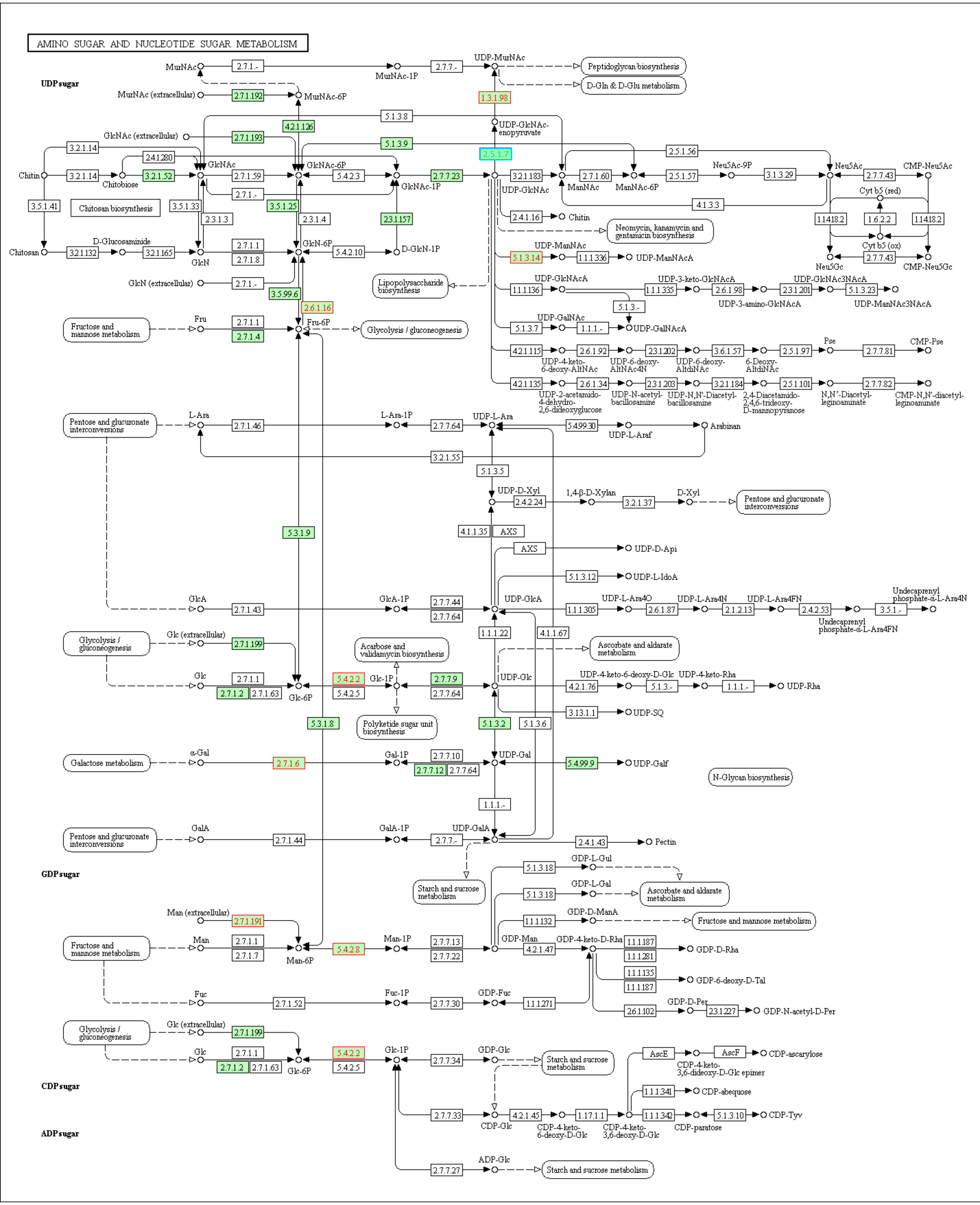

AMINO SUGAR AND NUCLEOTIDE SUGAR METABOLISM
UDP sugar
GDP sugar
CDP sugar
ADP sugar
MurNAc
2.7.1.-
MurNAc-1P
2.7.7.-
UDP-MurNAc
Peptidoglycan biosynthesis
D-Gln & D-Glu metabolism
1.3.1.98
MurNAc (extracellular)
2.7.1.192
MurNAc-6P
4.2.1.126
5.1.3.8
UDP-GlcNAc-enopyruvate
GlcNAc (extracellular)
2.7.1.193
5.1.3.9
2.5.1.7
3.2.1.14
2.4.1.280
2.5.1.56
Chitin
3.2.1.52
Chitobiose
GlcNAc
2.7.1.59
GlcNAc-6P
5.4.2.3
2.7.7.23
GlcNAc-1P
3.2.1.183
UDP-GlcNAc
ManNAc
2.7.1.60
ManNAc-6P
2.5.1.57
Neu5Ac-9P
3.1.3.29
Neu5Ac
2.7.7.43
CMP-Neu5Ac
Cyt b5 (red)
4.1.3.3
3.5.1.41
Chitosan biosynthesis
3.5.1.33
3.5.1.25
2.3.1.3
2.3.1.4
2.3.1.157
2.4.1.16
Neomycin, kanamycin and gentamicin biosynthesis
1.14.18.2
1.6.2.2
Cyt b5 (ox)
Neu5Gc
CMP-Neu5Gc
Chitosan
3.2.1.132
D-Glucosaminide
3.2.1.165
GlcN
2.7.1.1
2.7.1.8
GlcN-6P
5.4.2.10
D-GlcN-1P
UDP-ManNAc
5.1.3.14
1.1.1.336
UDP-ManNAcA
GlcN (extracellular)
3.5.99.6
Lipopolysaccharide biosynthesis
UDP-GlcNAcA
1.1.1.136
UDP-3-keto-GlcNAcA
1.1.1.335
2.6.1.98
UDP-3-amino-GlcNAcA
2.3.1.201
UDP-GlcNAc3NAcA
5.1.3.23
UDP-ManNAc3NAcA
5.1.3.-
2.6.1.16
Fructose and mannose metabolism
Fru
2.7.1.4
Fru-6P
Glycolysis / gluconeogenesis
UDP-GalNAc
5.1.3.7
1.1.1.-
4.2.1.115
2.6.1.92
2.3.1.202
3.6.1.57
2.5.1.97
Pse
2.7.7.81
CMP-Pse
UDP-4-keto-6-deoxy-AltNAc
UDP-6-deoxy-AltNAc4N
UDP-6-deoxy-AltdiNAc
6-Deoxy-AltdiNAc
4.2.1.135
2.6.1.34
2.3.1.203
3.2.1.184
2.5.1.101
2.7.7.82
UDP-2-acetamido-4-dehydro-2,6-dideoxyglucose
UDP-N-acetyl-bacillosamine
UDP-N,N'-Diacetyl-bacillosamine
2,4-Diacetamido-2,4,6-trideoxy-D-mannopyranose
N,N'-Diacetyl-legionaminate
CMP-N,N'-diacetyl-legionaminate
Pentose and glucuronate interconversions
L-Ara
2.7.1.46
L-Ara-1P
2.7.7.64
UDP-L-Ara
5.4.99.30
UDP-L-Araf
Arabinan
3.2.1.55
5.1.3.5
UDP-D-Xyl
2.4.2.24
1,4-β-D-Xylan
3.2.1.37
D-Xyl
5.3.1.9
4.1.1.35
AXS
UDP-D-Api
5.1.3.12
UDP-L-IdoA
GlcA
2.7.1.43
GlcA-1P
2.7.7.44
UDP-GlcA
1.1.1.305
UDP-L-Ara4O
2.6.1.87
UDP-L-Ara4N
2.1.2.13
UDP-L-Ara4FN
2.4.2.53
Undecaprenyl phosphate-α-L-Ara4FN
3.5.1.-
Undecaprenyl phosphate-α-L-Ara4N
1.1.1.22
4.1.1.67
Glc (extracellular)
2.7.1.199
Acarbose and validamycin biosynthesis
Ascorbate and aldarate metabolism
Glc
2.7.1.2
2.7.1.63
Glc-6P
5.4.2.2
5.4.2.5
Glc-1P
2.7.7.9
UDP-Glc
UDP-4-keto-6-deoxy-D-Glc
UDP-4-keto-Rha
4.2.1.76
UDP-Rha
3.13.1.1
UDP-SQ
5.3.1.8
Polyketide sugar unit biosynthesis
5.1.3.2
5.1.3.6
Galactose metabolism
α-Gal
2.7.1.6
Gal-1P
2.7.7.10
2.7.7.12
UDP-Gal
5.4.99.9
UDP-Galf
N-Glycan biosynthesis
GalA
2.7.1.44
GalA-1P
UDP-GalA
2.4.1.43
Pectin
5.1.3.18
GDP-L-Gul
GDP-L-Gal
Starch and sucrose metabolism
Man (extracellular)
2.7.1.191
1.1.1.132
GDP-D-ManA
Man
2.7.1.7
Man-6P
5.4.2.8
Man-1P
2.7.7.13
2.7.7.22
GDP-Man
4.2.1.47
GDP-4-keto-D-Rha
1.1.1.187
1.1.1.281
GDP-D-Rha
1.1.1.135
GDP-6-deoxy-D-Tal
Fuc
2.7.1.52
Fuc-1P
2.7.7.30
GDP-Fuc
1.1.1.271
2.6.1.102
GDP-D-Per
2.3.1.227
GDP-N-acetyl-D-Per
2.7.7.34
GDP-Glc
AscE
AscF
CDP-ascarylose
CDP-4-keto-3,6-dideoxy-D-Glc epimer
1.1.1.341
CDP-abequose
2.7.7.33
CDP-Glc
4.2.1.45
CDP-4-keto-6-deoxy-D-Glc
1.17.1.1
CDP-4-keto-3,6-deoxy-D-Glc
1.1.1.342
CDP-paratose
5.1.3.10
CDP-Tyv
2.7.7.27
ADP-Glc

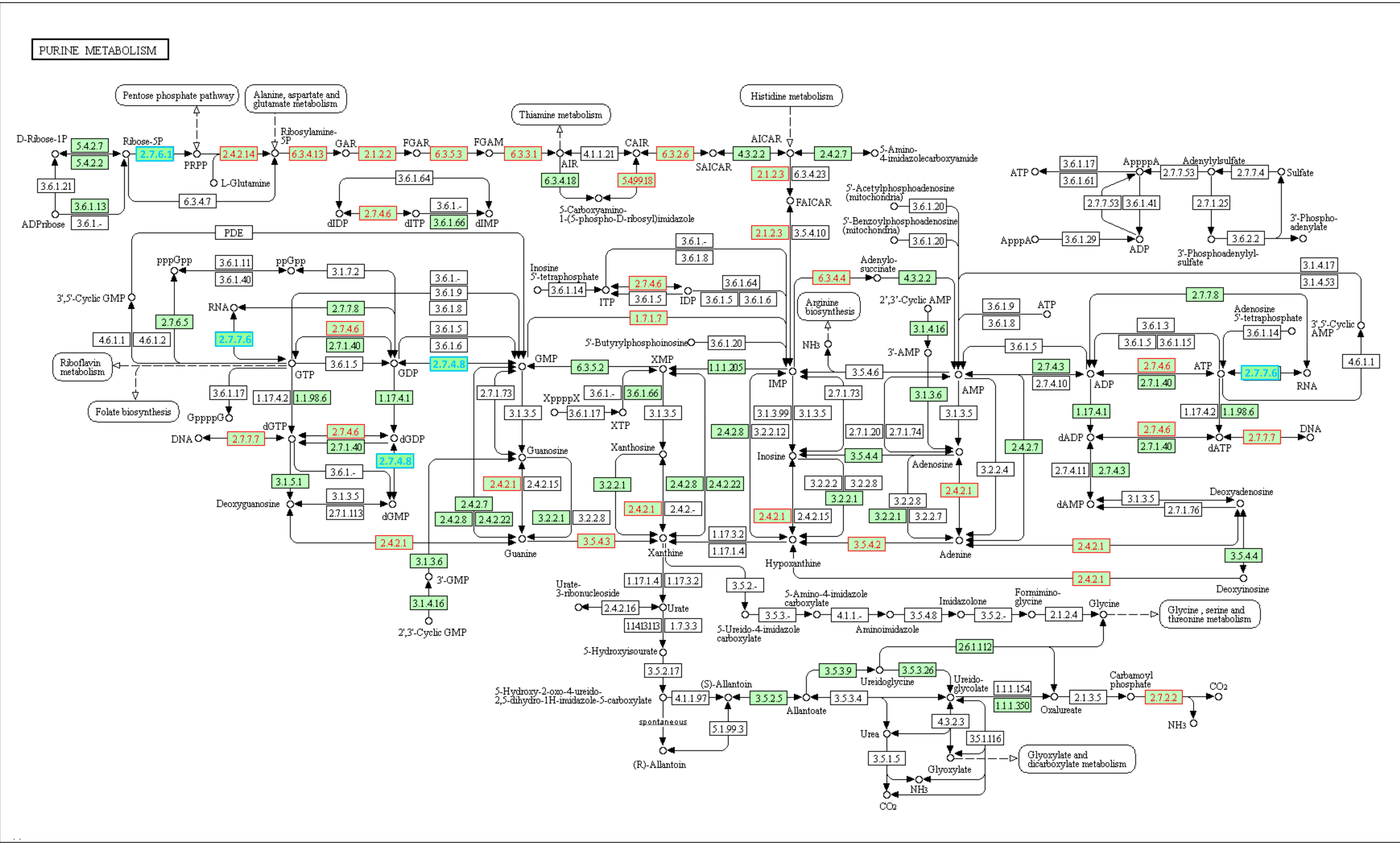
PURINE METABOLISM
Pentose phosphate pathway
Alanine, aspartate and glutamate metabolism
Thiamine metabolism
Histidine metabolism
D-Ribose-1P
Ribose-5P
PRPP
L-Glutamine
ADPribose
Ribosylamine-5P
GAR
FGAR
FGAM
AIR
CAIR
SAICAR
AICAR
5-Amino-4-imidazolecarboxyamide
5-Carboxyamino-1-(5-phospho-D-ribosyl)imidazole
FAICAR
dIDP
dITP
dIMP
PDE
pppGpp
ppGpp
3',5'-Cyclic GMP
RNA
Riboflavin metabolism
Folate biosynthesis
GppppG
GTP
GDP
GMP
dGTP
dGDP
dGMP
DNA
Deoxyguanosine
Guanosine
Guanine
3'-GMP
2',3'-Cyclic GMP
Inosine 5'-tetraphosphate
ITP
IDP
IMP
XMP
XppppX
XTP
Xanthosine
Xanthine
5'-Butyrylphosphoinosine
Inosine
Hypoxanthine
Adenylo-succinate
Arginine biosynthesis
NH3
2',3'-Cyclic AMP
3'-AMP
AMP
Adenosine
Adenine
ATP
ApppA
Adenylylsulfate
Sulfate
ADP
AppppA
5'-Acetylphosphoadenosine (mitochondria)
5'-Benzoylphosphoadenosine (mitochondria)
3'-Phospho-adenylate
3'-Phosphoadenylyl-sulfate
Adenosine 5'-tetraphosphate
3',5'-Cyclic AMP
dADP
dATP
dAMP
Deoxyadenosine
Deoxyinosine
Urate-3-ribonucleoside
Urate
5-Hydroxyisourate
5-Hydroxy-2-oxo-4-ureido-2,5-dihydro-1H-imidazole-5-carboxylate
spontaneous
(R)-Allantoin
(S)-Allantoin
Allantoate
5-Ureido-4-imidazole carboxylate
5-Amino-4-imidazole carboxylate
Aminoimidazole
Imidazolone
Formimino-glycine
Glycine
Glycine, serine and threonine metabolism
Ureidoglycine
Ureido-glycolate
Oxalureate
Carbamoyl phosphate
CO2
Urea
Glyoxylate
Glyoxylate and dicarboxylate metabolism

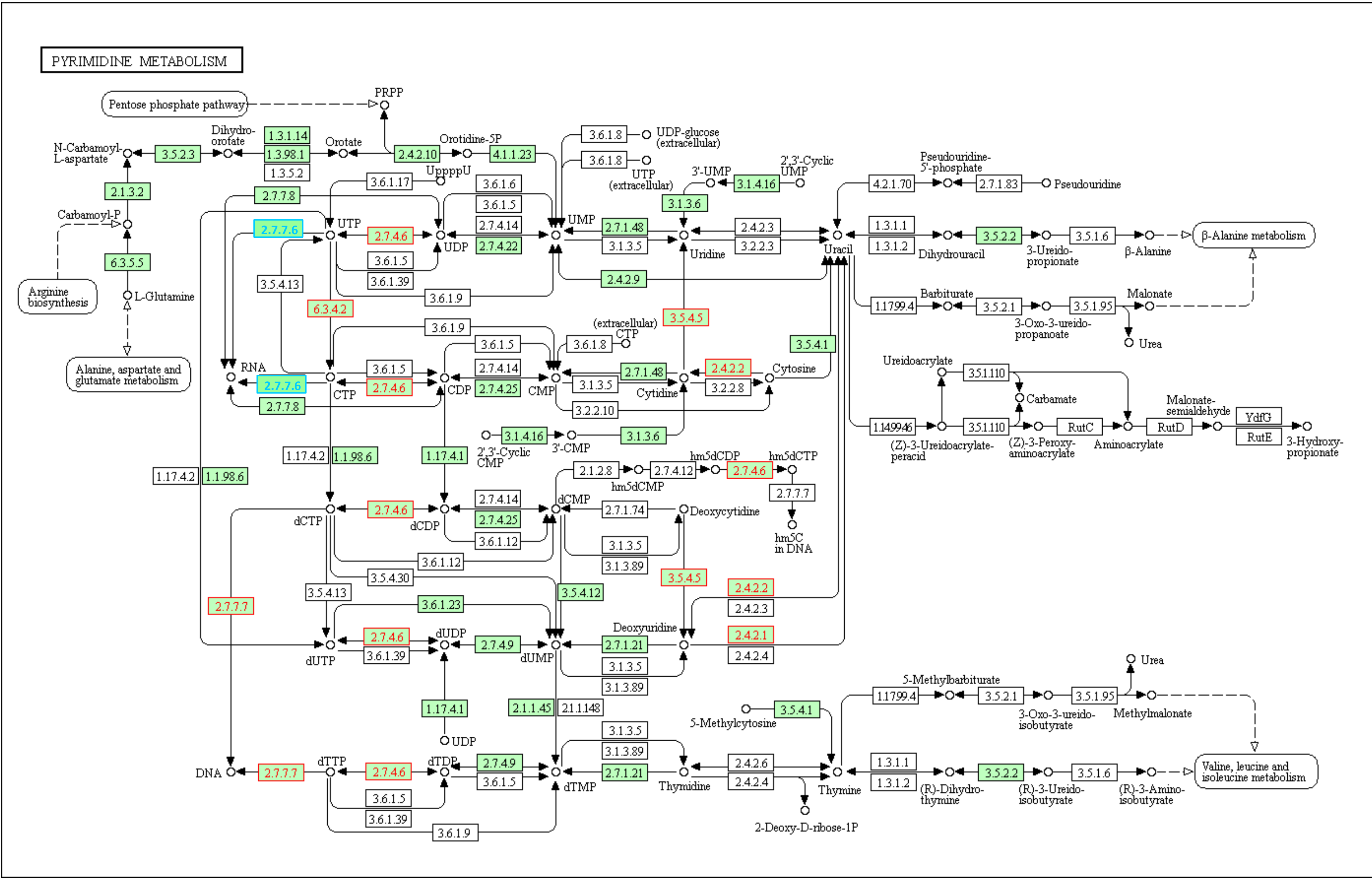
PYRIMIDINE METABOLISM
Pentose phosphate pathway
PRPP
N-Carbamoyl-L-aspartate
Dihydro-orotate
Orotate
Orotidine-5P
Carbamoyl-P
L-Glutamine
Arginine biosynthesis
Alanine, aspartate and glutamate metabolism
UTP
UDP
UMP
UppppU
RNA
CTP
CDP
CMP
UDP-glucose (extracellular)
UTP (extracellular)
3'-UMP
2',3'-Cyclic UMP
Uridine
Uracil
Cytidine
Cytosine
(extracellular) CTP
2',3'-Cyclic CMP
3'-CMP
Pseudouridine-5'-phosphate
Pseudouridine
Dihydrouracil
3-Ureido-propionate
β-Alanine
β-Alanine metabolism
Barbiturate
3-Oxo-3-ureido-propanoate
Malonate
Urea
Ureidoacrylate
Carbamate
(Z)-3-Ureidoacrylate-peracid
(Z)-3-Peroxy-aminoacrylate
Aminoacrylate
Malonate-semialdehyde
3-Hydroxy-propionate
dCTP
dCDP
dCMP
Deoxycytidine
hm5dCMP
hm5dCDP
hm5dCTP
hm5C in DNA
dUTP
dUDP
dUMP
Deoxyuridine
DNA
dTTP
dTDP
dTMP
Thymidine
Thymine
2-Deoxy-D-ribose-1P
5-Methylcytosine
5-Methylbarbiturate
3-Oxo-3-ureido-isobutyrate
Methylmalonate
(R)-Dihydro-thymine
(R)-3-Ureido-isobutyrate
(R)-3-Amino-isobutyrate
Valine, leucine and isoleucine metabolism
3.5.2.3
1.3.1.14
1.3.98.1
1.3.5.2
2.4.2.10
4.1.1.23
2.1.3.2
6.3.5.5
2.7.7.8
2.7.7.6
2.7.4.6
3.6.1.17
3.6.1.6
3.6.1.5
3.6.1.39
3.6.1.9
2.7.4.14
2.7.4.22
3.6.1.8
3.1.4.16
3.1.3.6
2.7.1.48
3.1.3.5
2.4.2.3
3.2.2.3
2.4.2.9
3.5.4.13
6.3.4.2
3.5.4.5
2.4.2.2
3.2.2.8
3.5.4.1
2.7.4.25
3.2.2.10
4.2.1.70
2.7.1.83
1.3.1.1
1.3.1.2
3.5.2.2
3.5.1.6
1.17.99.4
3.5.2.1
3.5.1.95
3.5.1.110
1.14.99.46
RutC
RutD
YdfG
RutE
1.17.4.2
1.1.98.6
1.17.4.1
2.1.2.8
2.7.4.12
2.7.7.7
2.7.1.74
3.6.1.12
3.1.3.89
3.5.4.30
3.5.4.12
3.6.1.23
2.4.2.1
2.4.2.4
2.7.4.9
2.7.1.21
2.1.1.45
2.1.1.148
2.4.2.6

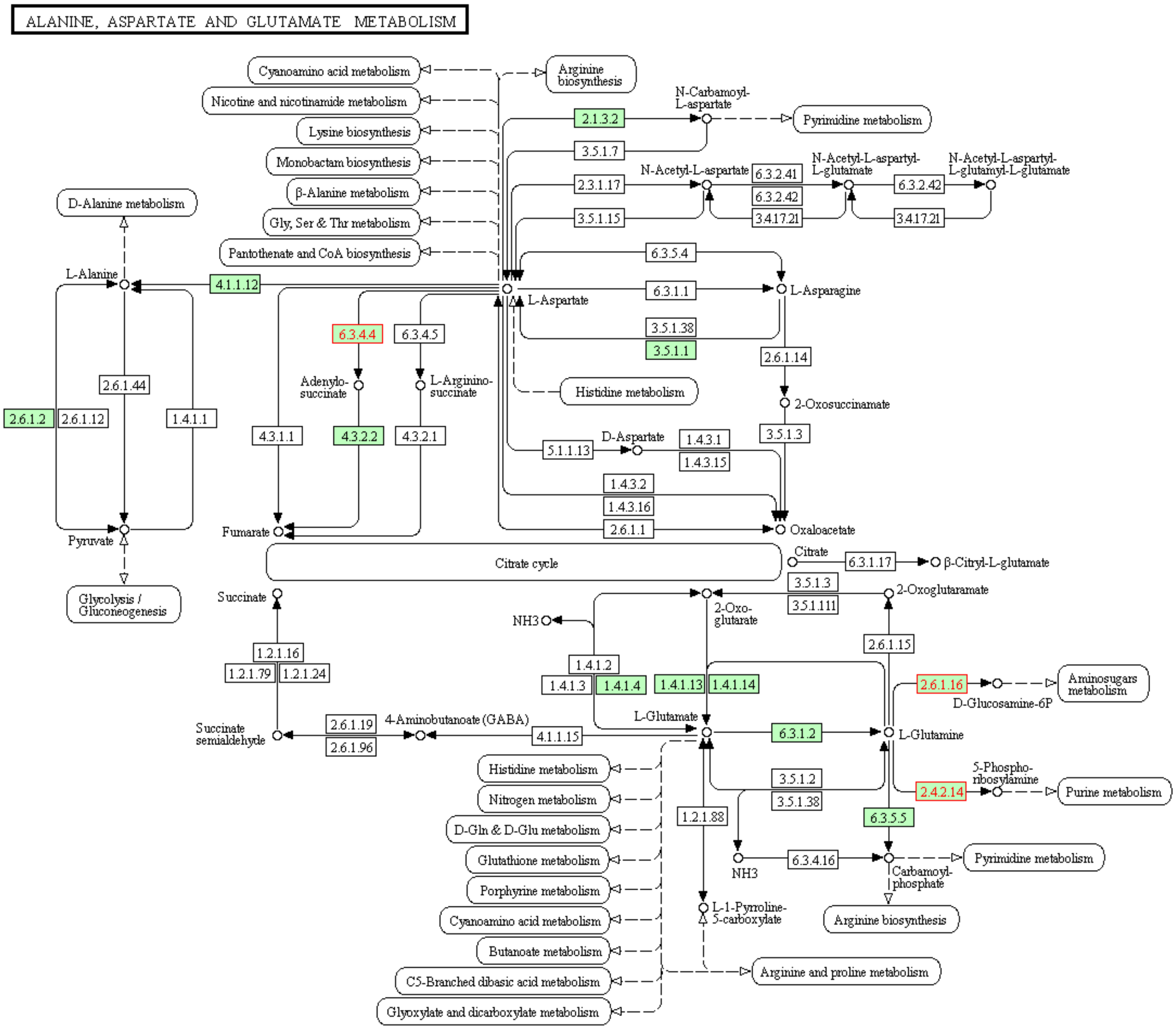
ALANINE, ASPARTATE AND GLUTAMATE METABOLISM
Cyanoamino acid metabolism
Nicotine and nicotinamide metabolism
Lysine biosynthesis
Monobactam biosynthesis
β-Alanine metabolism
Gly, Ser & Thr metabolism
Pantothenate and CoA biosynthesis
Arginine biosynthesis
N-Carbamoyl-L-aspartate
Pyrimidine metabolism
2.1.3.2
3.5.1.7
2.3.1.17
3.5.1.15
N-Acetyl-L-aspartate
6.3.2.41
6.3.2.42
3.4.17.21
N-Acetyl-L-aspartyl-L-glutamate
N-Acetyl-L-aspartyl-L-glutamyl-L-glutamate
D-Alanine metabolism
L-Alanine
4.1.1.12
L-Aspartate
6.3.5.4
6.3.1.1
L-Asparagine
3.5.1.38
3.5.1.1
6.3.4.4
6.3.4.5
Adenylo-succinate
L-Argininosuccinate
2.6.1.44
2.6.1.2
2.6.1.12
1.4.1.1
4.3.1.1
4.3.2.2
4.3.2.1
Histidine metabolism
2.6.1.14
2-Oxosuccinamate
3.5.1.3
5.1.1.13
D-Aspartate
1.4.3.1
1.4.3.15
1.4.3.2
1.4.3.16
2.6.1.1
Pyruvate
Fumarate
Oxaloacetate
Glycolysis / Gluconeogenesis
Citrate cycle
Citrate
6.3.1.17
β-Citryl-L-glutamate
3.5.1.3
3.5.1.111
2-Oxoglutaramate
Succinate
NH3
2-Oxo-glutarate
2.6.1.15
1.2.1.16
1.2.1.79
1.2.1.24
1.4.1.2
1.4.1.3
1.4.1.4
1.4.1.13
1.4.1.14
2.6.1.16
Aminosugars metabolism
D-Glucosamine-6P
Succinate semialdehyde
2.6.1.19
2.6.1.96
4-Aminobutanoate (GABA)
4.1.1.15
L-Glutamate
6.3.1.2
L-Glutamine
5-Phospho-ribosylamine
2.4.2.14
Purine metabolism
Histidine metabolism
Nitrogen metabolism
D-Gln & D-Glu metabolism
Glutathione metabolism
Porphyrine metabolism
Cyanoamino acid metabolism
Butanoate metabolism
C5-Branched dibasic acid metabolism
Glyoxylate and dicarboxylate metabolism
3.5.1.2
3.5.1.38
1.2.1.88
6.3.5.5
NH3
6.3.4.16
Carbamoyl-phosphate
Pyrimidine metabolism
L-1-Pyrroline-5-carboxylate
Arginine biosynthesis
Arginine and proline metabolism

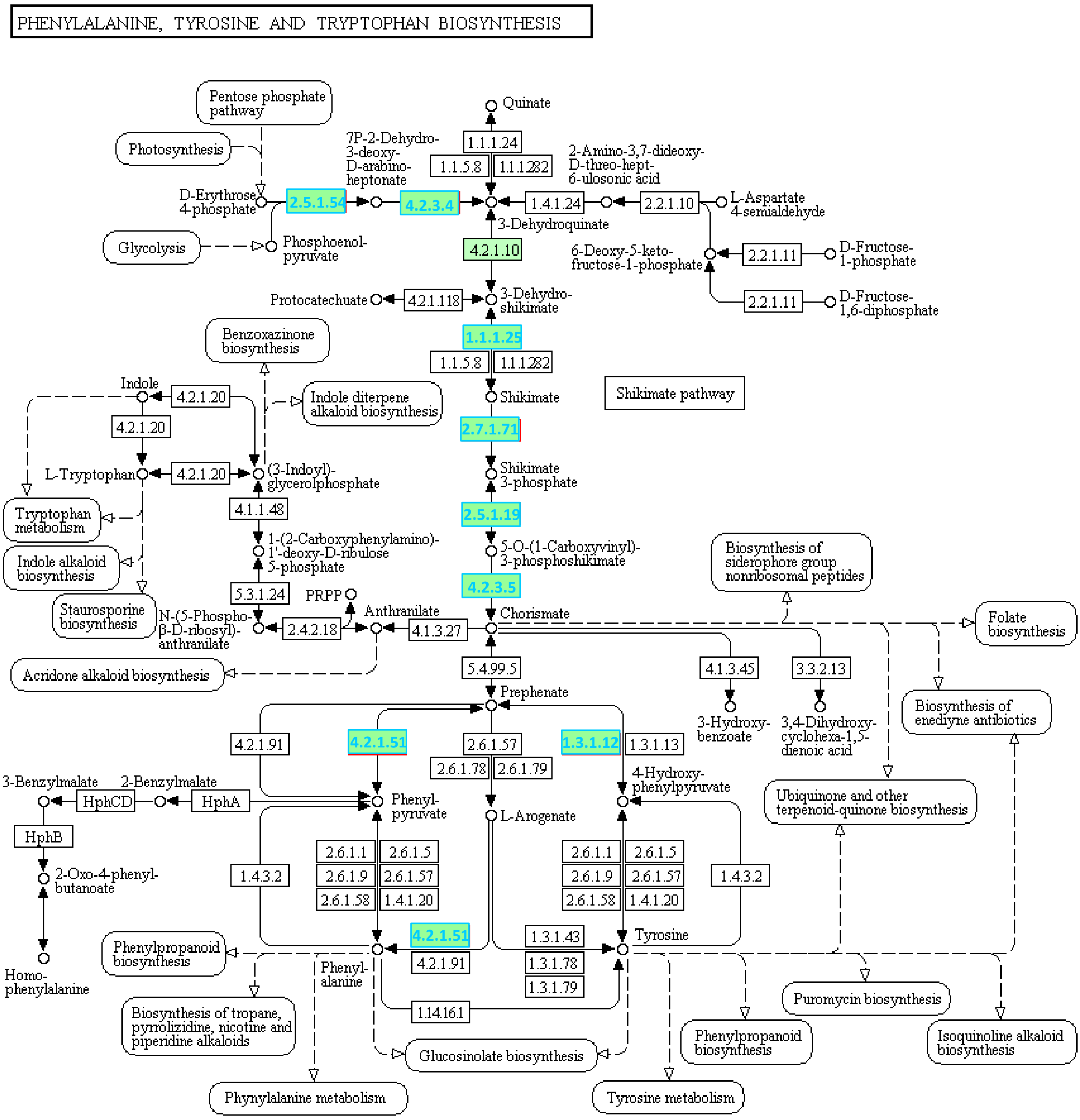
PHENYLALANINE, TYROSINE AND TRYPTOPHAN BIOSYNTHESIS
Pentose phosphate pathway
Photosynthesis
Glycolysis
D-Erythrose 4-phosphate
2.5.1.54
7P-2-Dehydro-3-deoxy-D-arabino-heptonate
4.2.3.4
Quinate
1.1.1.24
1.1.5.8
1.1.1.282
2-Amino-3,7-dideoxy-D-threo-hept-6-ulosonic acid
1.4.1.24
2.2.1.10
L-Aspartate 4-semialdehyde
3-Dehydroquinate
Phosphoenol-pyruvate
4.2.1.10
6-Deoxy-5-keto-fructose-1-phosphate
2.2.1.11
D-Fructose-1-phosphate
D-Fructose-1,6-diphosphate
Protocatechuate
4.2.1.118
3-Dehydro-shikimate
1.1.1.25
Benzoxazinone biosynthesis
Shikimate
Shikimate pathway
Indole
4.2.1.20
Indole diterpene alkaloid biosynthesis
2.7.1.71
L-Tryptophan
(3-Indoyl)-glycerolphosphate
Shikimate 3-phosphate
Tryptophan metabolism
4.1.1.48
2.5.1.19
1-(2-Carboxyphenylamino)-1'-deoxy-D-ribulose 5-phosphate
5-O-(1-Carboxyvinyl)-3-phosphoshikimate
Indole alkaloid biosynthesis
Biosynthesis of siderophore group nonribosomal peptides
5.3.1.24
PRPP
4.2.3.5
Staurosporine biosynthesis
N-(5-Phospho-β-D-ribosyl)-anthranilate
2.4.2.18
Anthranilate
4.1.3.27
Chorismate
Folate biosynthesis
Acridone alkaloid biosynthesis
5.4.99.5
4.1.3.45
3.3.2.13
Prephenate
3-Hydroxy-benzoate
3,4-Dihydroxy-cyclohexa-1,5-dienoic acid
Biosynthesis of enediyne antibiotics
4.2.1.91
4.2.1.51
2.6.1.57
1.3.1.12
1.3.1.13
2.6.1.78
2.6.1.79
4-Hydroxy-phenylpyruvate
3-Benzylmalate
2-Benzylmalate
HphCD
HphA
Phenyl-pyruvate
L-Arogenate
Ubiquinone and other terpenoid-quinone biosynthesis
HphB
2.6.1.1
2.6.1.5
2.6.1.9
2.6.1.57
2.6.1.58
1.4.1.20
1.4.3.2
2-Oxo-4-phenyl-butanoate
1.3.1.43
Tyrosine
1.3.1.78
1.3.1.79
Phenylpropanoid biosynthesis
Phenyl-alanine
Homo-phenylalanine
1.14.16.1
Puromycin biosynthesis
Biosynthesis of tropane, pyrrolizidine, nicotine and piperidine alkaloids
Glucosinolate biosynthesis
Isoquinoline alkaloid biosynthesis
Phynylalanine metabolism
Tyrosine metabolism

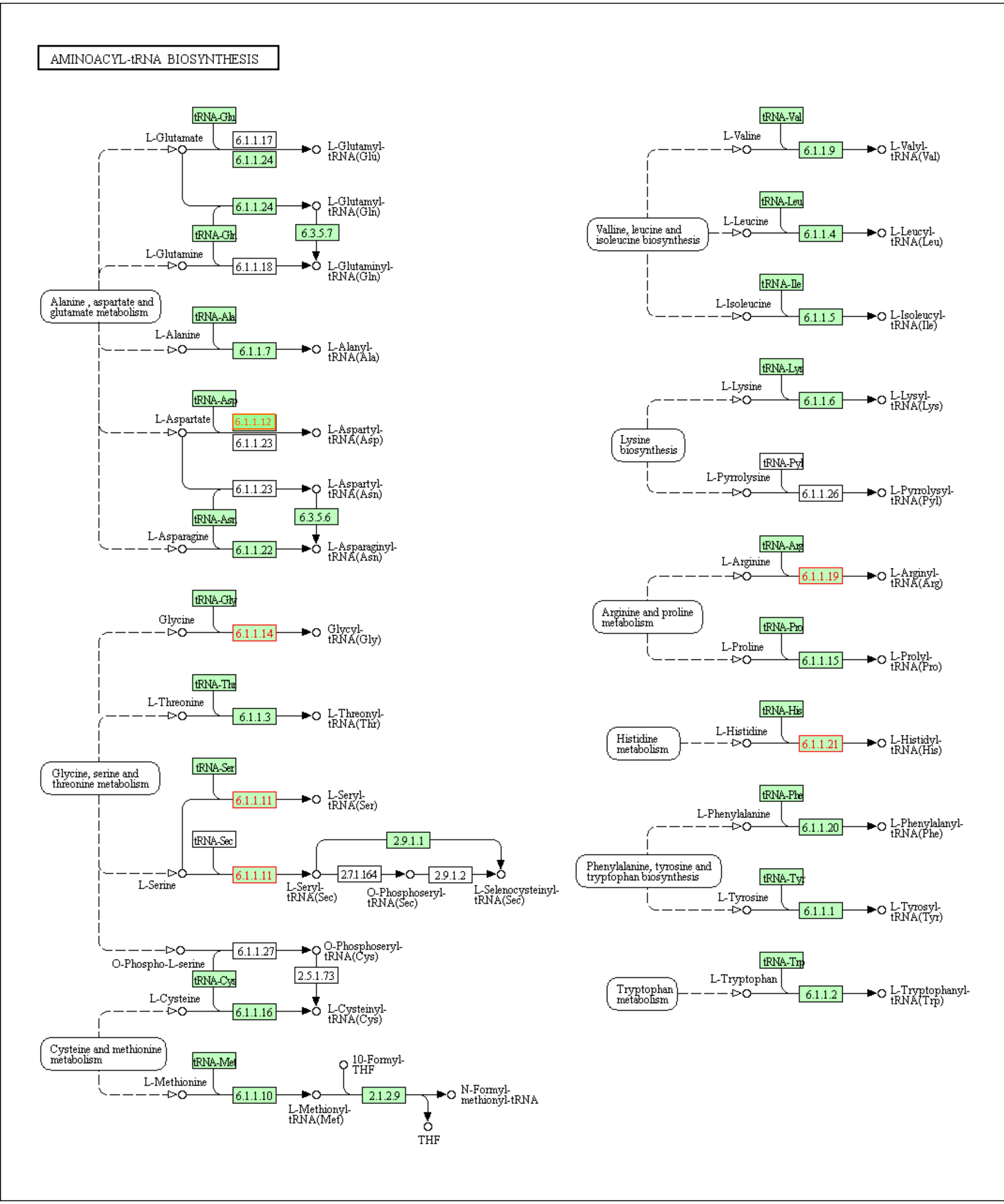

AMINOACYL-tRNA BIOSYNTHESIS
tRNA-Glu
L-Glutamate
6.1.1.17
6.1.1.24
L-Glutamyl-tRNA(Glu)
6.1.1.24
L-Glutamyl-tRNA(Gln)
tRNA-Gln
6.3.5.7
L-Glutamine
6.1.1.18
L-Glutaminyl-tRNA(Gln)
Alanine , aspartate and glutamate metabolism
tRNA-Ala
L-Alanine
6.1.1.7
L-Alanyl-tRNA(Ala)
tRNA-Asp
L-Aspartate
6.1.1.12
6.1.1.23
L-Aspartyl-tRNA(Asp)
6.1.1.23
L-Aspartyl-tRNA(Asn)
tRNA-Asn
6.3.5.6
L-Asparagine
6.1.1.22
L-Asparaginyl-tRNA(Asn)
tRNA-Gly
Glycine
6.1.1.14
Glycyl-tRNA(Gly)
tRNA-Thr
L-Threonine
6.1.1.3
L-Threonyl-tRNA(Thr)
Glycine, serine and threonine metabolism
tRNA-Ser
6.1.1.11
L-Seryl-tRNA(Ser)
tRNA-Sec
2.9.1.1
L-Serine
6.1.1.11
L-Seryl-tRNA(Sec)
2.7.1.164
O-Phosphoseryl-tRNA(Sec)
2.9.1.2
L-Selenocysteinyl-tRNA(Sec)
O-Phospho-L-serine
6.1.1.27
O-Phosphoseryl-tRNA(Cys)
2.5.1.73
tRNA-Cys
L-Cysteine
6.1.1.16
L-Cysteinyl-tRNA(Cys)
Cysteine and methionine metabolism
tRNA-Met
L-Methionine
6.1.1.10
L-Methionyl-tRNA(Met)
10-Formyl-THF
2.1.2.9
N-Formyl-methionyl-tRNA
THF
tRNA-Val
L-Valine
6.1.1.9
L-Valyl-tRNA(Val)
Valline, leucine and isoleucine biosynthesis
tRNA-Leu
L-Leucine
6.1.1.4
L-Leucyl-tRNA(Leu)
tRNA-Ile
L-Isoleucine
6.1.1.5
L-Isoleucyl-tRNA(Ile)
tRNA-Lys
L-Lysine
6.1.1.6
L-Lysyl-tRNA(Lys)
Lysine biosynthesis
tRNA-Pyl
L-Pyrrolysine
6.1.1.26
L-Pyrrolysyl-tRNA(Pyl)
tRNA-Arg
L-Arginine
6.1.1.19
L-Arginyl-tRNA(Arg)
Arginine and proline metabolism
tRNA-Pro
L-Proline
6.1.1.15
L-Prolyl-tRNA(Pro)
tRNA-His
Histidine metabolism
L-Histidine
6.1.1.21
L-Histidyl-tRNA(His)
tRNA-Phe
L-Phenylalanine
6.1.1.20
L-Phenylalanyl-tRNA(Phe)
Phenylalanine, tyrosine and tryptophan biosynthesis
tRNA-Tyr
L-Tyrosine
6.1.1.1
L-Tyrosyl-tRNA(Tyr)
tRNA-Trp
Tryptophan metabolism
L-Tryptophan
6.1.1.2
L-Tryptophanyl-tRNA(Trp)
